# Oxygenation of Hypoxic Coastal Baltic Sea Sediments Impacts on Chemistry, Microbial Community Composition, and Metabolism

**DOI:** 10.3389/fmicb.2017.02453

**Published:** 2017-12-12

**Authors:** Elias Broman, Varvara Sachpazidou, Jarone Pinhassi, Mark Dopson

**Affiliations:** Biology and Environmental Sciences, Centre for Ecology and Evolution in Microbial Model Systems, Linnaeus University, Kalmar, Sweden

**Keywords:** 16S rRNA, anoxic, oxic, sulfur, metatranscriptomics, sediment

## Abstract

The Baltic Sea has undergone severe eutrophication during the last century, resulting in increased algal blooms and the development of hypoxic bottom waters. In this study, we sampled oxygen deficient sediment cores from a Baltic Sea coastal bay and exposed the bottom water including the sediment surface to oxygen shifts via artificial addition of air during laboratory incubation. Surface sediment (top 1 cm) from the replicate cores were sliced in the field as well as throughout the laboratory incubations and chemical parameters were analyzed along with high throughput sequencing of community DNA and RNA. After oxygenation, dissolved iron decreased in the water overlying the sediment while inorganic sulfur compounds (thiosulfate and tetrathionate) increased when the water was kept anoxic. Oxygenation of the sediment also maintained RNA transcripts attributed to sulfide and sulfur oxidation as well as nitrogen fixation in the sediment surface. Based on 16S rRNA gene and metatranscriptomic analyses it was found that oxygenation of the sediment surface caused a bloom of the *Epsilonproteobacteria* genus *Arcobacter*. In addition, the formation of a thick white film was observed that was likely filamentous zero-valent sulfur produced by the *Arcobacter* spp. Based on these results, sulfur cycling and nitrogen fixation that were evident in the field samples were ongoing during re-oxygenation of the sediment. These processes potentially added organic nitrogen to the system and facilitated the re-establishment of micro- and macroorganism communities in the benthic zone.

## Introduction

The Baltic Sea has undergone severe eutrophication during the last century (Conley et al., 2002; Carstensen et al., 2014) and the resulting increase in nutrient input fuels phytoplankton blooms which eventually die and sink to the sediment surface (Emeis et al., 2000). This organic matter is degraded by microorganisms and the surface layers of the sediment quickly become oxygen deficient for aerobic micro- and macroinvertebrates (Middelburg and Meysman, 2007). These hypoxic (<2 O_2_ mg/L) and anoxic areas, coined as “dead zones” (Conley, 2012), are widespread in coastal areas of the Baltic Sea (Conley et al., 2011). Anoxia in the sediment surface favors anaerobic microorganisms (Nealson, 1997; Burdige, 2006; Köchling et al., 2011) able to reduce ferric iron (Fe^3+^) and sulfate (${\text{SO}}_{4}^{2-}$) to ferrous iron (Fe^2+^) and the toxic gas hydrogen sulfide (H_2_S), respectively (Bagarinao, 1992; Burdige, 2006). These processes result in oxygen deficient sediments having a characteristic black color due to iron sulfides produced from the coupling of Fe^2+^ and H_2_S (Burdige, 2006), and early hypoxia development favors anaerobic microbes responsible for denitrification (Conley and Johnstone, 1995; Tuominen et al., 1999). In contrast, it is unknown what metabolic functions are favored during the initial stages of anoxic sediment re-oxygenation.

Anaerobic microorganisms in the sediment thrive using the electron acceptors (in the order of decreasing energy gain): nitrate (${\text{NO}}_{3}^{-}$), manganese (Mn^3+^ and Mn^4+^) oxides, Fe^3+^ oxides, and ${\text{SO}}_{4}^{2-}$. Due to the different energy efficiency of the electron acceptors, a redox cascade is formed with the most energy yielding electron acceptor being utilized first (Burdige, 2006). Additionally, methane (CH_4_) is produced (i.e., methanogenesis) by microbial reduction of carbon dioxide (CO_2_) and organic matter is fermented below the ${\text{SO}}_{4}^{2-}$ reduction zone (Burdige, 2006). The depth of this redox cascade below the sediment surface depends on the organic matter content and can occur within just a few millimeters (Burdige, 1993; Jørgensen, 2006; Middelburg and Meysman, 2007) or deeper in the sediments. For instance, methane production can occur in the 0-20 mm layer (Xiao et al., 2017) as well as below this layer. The reduced products (e.g., Mn^2+^, Fe^2+^, and S^2−^) diffuse upwards where they can be oxidized by oxygen (chemical or microbial oxidation) or be utilized in other anaerobic redox processes such as denitrification (Straub et al., 1996; Burdige, 2006). Variation in the concentrations of electron acceptors influences the location of the redox zones; for example, anoxic conditions in the bottom water induces a ${\text{SO}}_{4}^{2-}$ reduction zone in the sediment surface (Burdige, 2006). Currently, the time frame for changes in microbial metabolic functions in the redox zones during re-oxygenation of anoxic sediments is unknown.

Microbial communities in marine sediments are rich in sulfate reducing *Delta-* and *Gammaproteobacteria* and these communities have been well studied in oxygen rich, hypoxic, and anoxic sites (e.g., Edlund et al., 2006; Swan et al., 2010; Divya et al., 2011; Köchling et al., 2011; Alneberg et al., 2014). A Baltic Sea transect study of microbial populations in the sediment surface with varying oxygen levels found that classes within the *Proteobacteria* fluctuated depending on oxygen availability (Edlund et al., 2006). Differences in microbial communities in oxygen rich and anoxic sites have been further verified, with changes occurring in e.g., *Spirochaetes, Delta-*, and *Epsilonproteobacteria* (Broman et al., 2017a). In addition, Thureborn et al. (2016) found that dominant processes in the deepest anoxic sediment in the Baltic Sea were related to organic matter degradation, sulfate reduction, and methane production. Even though hypoxia/anoxia is the key characteristic of “dead zones,” it is not well understood how the interplay between fluxes in chemical parameters and microbial metabolic functions are influenced during natural oxygenation events.

Inflow of oxygen rich water can oxygenate anoxic bottom zones in the Baltic Sea (Rosenberg et al., 2016) and the extent of these zones can therefore, increase or decrease over time (Carstensen et al., 2014). A previous experiment by Broman et al. (2017a) showed that oxygenation of anoxic coastal Baltic Sea sediments in an incubation experiment for 21 days resulted in a proliferation in sulfide oxidizing *Epsilonproteobacteria* and higher RNA transcript counts for genes attributed to sulfide and methane oxidation. In addition, a recent study by Lipsewers et al. (2017) found that in a closed coastal water system, sulfide oxidizing *Epsilonproteobacteria* were stimulated during oxic conditions in spring, while the community composition shifted to favor sulfate reducing *Deltaproteobacteria* during anoxic conditions during autumn. However, the effect on the microbial community structure and metabolism during the first few days of oxygenation remains unknown.

In this study, we collected black, H_2_S-rich coastal Baltic Sea sediments from a site with bottom waters with near hypoxic conditions. The impact of altering oxygen concentrations was investigated by artificially oxygenating the benthic water overlying the sediment. The aims of the study were: (1) to identify alterations in the bottom water and sediment microbial community; (2) to follow microbial process changes in the sediment surface at the start, during, and at the end of the oxygenation experiment; and (3) to link these findings to measured chemistry fluxes.

## Materials and methods

### Sampling of sediment cores

Sediment (31 m below the sea surface) was sampled on the 15 April 2016 from a Baltic Sea coastal bay near the town Loftahammar (WGS 84 coordinates: Lat. 57 53.531, Lon. 16 35.165). Sampling procedures plus handling of water and sediments are described in Broman et al. (2017a). A total of 16 sediment cores with overlying benthic water were collected (in a radius of ~5 m). The cores contained a height of 34.0-41.6 cm sediment and 11.5–15.5 cm water phase. Previously the sampling site had undergone long-term anoxia (Broman et al., 2015), while on this occasion the sediment was black, with a strong odor of H_2_S, and white spots of microbial mats on the sediment surface indicating sulfide oxidation. The 11.5–15.5 cm bottom water column overlying the sediment in the cores had an oxygen concentration of 3.83 mg/L (*in situ* measurement using an oxygen sensor, WTW Multiline). As hypoxia is defined as <2 mg/L the bottom waters had a low oxygen concentration at the time of sampling. However, that the sediment was black with white spots indicated that the sediment was oxygen deficient. In three cores, water was sub-sampled from the water phase and the top 1 cm sediment layer was sliced in the field (Broman et al., 2017a). Collected water and sediment were divided and maintained at 4°C until chemical analysis of water and sediment pore-water nitrite (${\text{NO}}_{2}^{-}$)+${\text{NO}}_{3}^{-}$, ${\text{PO}}_{4}^{3-}$, ${\text{SO}}_{4}^{2-}$, total iron (Fe_tot_), tetrathionate (S_4_${\text{O}}_{6}^{2-}$), thiosulfate (S_2_${\text{O}}_{3}^{2-}$), pH, redox potential, and sediment organic matter % (Broman et al., 2017a). Additionally, water was collected for DNA extractions and the top 1 cm sediment slices were also stored for DNA and RNA extraction (Broman et al., 2017a). Samples for DNA extraction were kept on ice while those for RNA extraction were mixed with 5% water-saturated phenol in absolute ethanol (Feike et al., 2012) and then flash frozen in liquid nitrogen until being stored at −80°C after transport to the laboratory. A set of 13 sediment cores were then collected and closed at the bottom and top and brought back to the laboratory on the same day (maintained at ~9°C during transportation).

### Incubation of sediment cores

A detailed methodology of the sub-sampling, slicing, and incubation setup has been previously described (Broman et al., 2017a). After sampling, sediment cores were stored overnight at 8°C in darkness before the incubations were initiated. Ten cores were continuously aerated by bubbling air into the overlying water phase (designated “turned oxic”), while the other three cores were turned anoxic (designated “turned anoxic”) by initially bubbling N_2_-gas for 40 min and for 20 min during sampling at each time point. To ensure mixing of the water column, a sterile polytetrafluoroethylene tube (Sarstedt) containing neodym magnets was swirled in all cores by rotating external magnets. Sub-sampling of the water phase from all cores was conducted when the cores were sliced for sediment (i.e., sacrificed) on days 0, 5, 9, and 15 for chemistry measurements and DNA extraction. Oxygen concentrations were also measured in the water phase after sampling using an optical oxygen sensor (FireStingO2; OXR50 oxygen sensor, PyroScience). Sediment cores turned oxic were sacrificed at the various time points and the top 1 cm sediment layer was collected for chemistry as well as for DNA and RNA extraction. The cores turned anoxic were all sliced on day 15. In addition to slicing on these days, two oxygen profiles were measured in the sediment surface of the cores before slicing using the oxygen sensor connected to a 50 μm resolution micromanipulator (Micromanipulator MU1, PyroScience). Sub-sampling of water and slicing of sediment was conducted in the same manner as the field cores.

### Chemistry measurements

A full description of the methods and apparatus used for chemistry measurements has been published (Broman et al., 2017a). Sediment pore-water was collected by centrifuging sediment samples at 2,200 g for 15 min and the supernatant filtered through a 0.7 μm 30 mm diameter glass fiber filter (30-SF-07 GMF syringe filter, Chromacol). Water and sediment pore-water ${\text{NO}}_{2}^{-}+$${\text{NO}}_{3}^{-}$ and ${\text{PO}}_{4}^{3-}$ concentrations were measured spectrophotometrically according to modified protocols by Valderrama (1995) (detection limits of: 0.02–32 μM and 0.02–50 μM, respectively). ${\text{SO}}_{4}^{2-}$ was measured using the Hach-Lange LCK 353 kit (detection limits: 1.56–9.37 mM) and Fe_tot_ was measured using the ferrozine method (detection limits: 0.3–100 μM) according to Dawson and Lyle (1990), with the exception that samples were incubated for 1 h in darkness during ferrozine reaction. Tetrathionate and thiosulfate were analyzed by cyanolysis (Sörbo, 1957) according to Kelly et al. (1969) (detection limits 0.4 μM to 1 mM for both). pH and redox potential were analyzed using electrodes (pHenomenal, VWR pH electrode and Ag^0^/AgCl SI Analytics electrode, Mettler Toledo; respectively). The percentage of organic matter in the sediment surface was determined by loss on ignition of dry sediment (Broman et al., 2017a).

### DNA and RNA extraction, sequencing, and bioinformatic analysis

DNA from frozen water filters, DNA from frozen sediment, and RNA from frozen sediment mixed with fix-solution were extracted using the PowerWater DNA, PowerSoil DNA, and PowerSoil RNA kits (MO BIO Laboratories), respectively. DNA was removed from extracted RNA using the Turbo DNA-free kit (Ambion) and rRNA depletion was conducted with the Ribominus Transcriptome Isolation Kit (Bacteria version; Invitrogen Life Technologies). cDNA synthesis was conducted from the rRNA depleted samples using the Ovation RNA-Seq System V2 kit (NuGEN) and purified with the MinElute Reaction Cleanup kit (QIAGEN).

Water and sediment samples for DNA extraction were treated by propidium monoazide (PMA) according to Checinska et al. (2015), except that phosphate buffered saline (PBS) was used instead of water to prevent osmotic stress. PMA inhibits non-viable cells from amplifying during PCR by entering cells with a compromised membrane and binding to the DNA (Nocker et al., 2006). The PMA treatment was conducted before DNA extraction on cells captured from the water phase on a 0.22 μm filter by aseptically cutting the filters and placing them into 1 mL sterile filtered PBS (AMRESCO). PMA treatment on the sediment samples was carried out by adding PBS to 0.25 g sediment to make a total of 1 mL and adding 12.5 μL PMA (2 mM, Biotium, Inc., Hayward, CA; giving a final concentration of 25 μM PMA). Both sets of samples were then incubated in darkness for 5 min before UV irradiation for 15 min (PhAST blue-Photo activation, GenIUL, S.L., Terrassa, Spain). DNA from the total cells (i.e., both viable and non-viable cells) was extracted from the second half of the cut filter by the same procedure except that PMA was omitted. These two datasets were designated as the “total” and “viable” populations (i.e., all cells and cells with an intact membrane, respectively).

Preparation and sequencing of 16S rRNA gene amplicons were conducted as described in Broman et al. (2017a) at the Science for Life Laboratory, Sweden (www.scilifelab.se) using the MiSeq Illumina platform. Briefly, primers 341F and 805R (Herlemann et al., 2011) were used to amplify partial 16S rRNA gene sequences according to PCR programs by Hugerth et al. (2014) and library preparation was conducted as described in Lindh et al. (2015). These primers have previously been shown to favor the bacterial 16S rRNA gene over Archaea (Hugerth et al., 2014) although some archaeal sequences are amplified. 16S rRNA gene sequence data were processed using the UPARSE pipeline (Edgar, 2013) and clustered 16S rRNA gene operational taxonomical units (OTUs) at 97% sequence similarity were annotated against the small subunit SINA/SILVA database (Quast et al., 2013). Final OTU taxonomy tables were analyzed using the software Explicet (Robertson et al., 2013), and multivariate analysis was conducted in Past v 3.10 (Hammer et al., 2001). Rarefaction curves of OTUs, read counts, and the rarefied species richness were constructed in R using the vegan package. Alpha diversity was calculated using Shannon's H index on OTU counts sub-sampled to the lowest sample size (1776 counts) and bootstrapped 100 times. A correlation network of 16S rRNA gene OTUs was created in sparCC version 2016-10-17 (Friedman and Alm, 2012) and *p*-values determined from 100 permutations. Correlations with *r* < −0.7 or > 0.7 and with a *p* < 0.01 were visualized as a network in Cytoscape version 3.5.1 (Shannon et al., 2003).

cDNA samples were sequenced at the Science for Life Laboratory using the Illumina HiSeq platform (pair ends 2 × 126 bp). Sequences were trimmed for Illumina adapters with SeqPrep, quality trimmed with Trimmomatic (parameters: LEADING:5 TRAILING:5 MINLEN:36; Bolger et al., 2014), and yielded on average 16.4 million reads per sample with a length of 124 bp. The reads were then used to construct a *de novo* assembly using Trinity (Haas et al., 2013) yielding 431522 contigs with an average length of 319 bp. After assembly, the Trinotate pipeline (https://trinotate.github.io/) was used as a guideline. In more detail, TransDecoder 2.0.1 was use to find the longest open reading frames. Annotations of the assembled contigs were performed using BLASTX while annotation of the ORFs was carried out with BLASTP and hmmscan against the UniProtKB/Swiss-Prot and PFAM databases, respectively. Also, rRNA sequences were identified with RNAmmer 1.2. Reads were mapped back to assembled contigs to estimate counts of rRNA sequences using RSEM (Li and Dewey, 2011) in conjunction with samtools 1.165 (Li et al., 2009) and Bowtie2 2.2.3 (Langmead et al., 2009). Read counts among samples were expressed as Trimmed Mean of M-values (TMM; Robinson and Oshlack, 2010) normalized Fragment Per Kilobase Million reads (FPKM) values. FPKM was calculated as: (fragments ^*^ 1,000,000)/(length of transcript in kb ^*^ sum of all mapped reads). All expression values were calculated as default by following the Trinotate pipeline (https://trinotate.github.io/). RNA transcripts were grouped according to their UniProtKB/Swiss-Prot identifier (i.e., genes) and genes that could be directly linked to the Archaea and Bacteria Kingdom with a FPKM value higher than 1,000 were extracted for further analysis. Differential expression testing was conducted using the raw read counts in the R package edgeR (Robinson et al., 2010). Reference organisms in the UniProtKB/Swiss-Prot database linked to RNA transcripts of annotated genes were used to infer the active microbial community composition. A subset of these genes that could be linked to identified chemical processes in the sediments were categorized according to UniProtKB/Swiss-Prot, MetaCyc, KEGG Orthology, and GO biological terminology. Additionally, genes directly annotated to the genus *Arcobacter* in the UniProtKB/Swiss-Prot database were extracted and further analyzed as described above.

Information from the DNA and RNA sequence data analysis, such as the number of reads and contigs etc. is available in Supplemental Table 1. Bioinformatics were conducted using the UPPMAX Next Generation sequence Cluster Storage (UPPNEX) projects b2013127 and b2016308 at the Uppsala Multidisciplinary Center for Advanced Computational Science (UPPMAX). The 16S rRNA gene sequences and metatranscriptomic sequence data have been uploaded to the NCBI database and have the BioProject accession number: PRJNA347538.

## Results

### Chemical fluxes in the water phase and sediment

The oxygen concentrations in the water phase of the “turned-oxic” sediment cores were ~ 10 mg/L throughout the incubation experiment, while the “turned anoxic” cores ranged between 0.00 and 0.07 mg/L. A full list of all chemistry measurements in the water phase at the start of the experiment and for each sub-sampling can be found in Supplemental Table 2 (oxygen data from Broman et al., 2017b). Chemistry measurements conducted in the field showed a surface water temperature of 5.7°C, pH 8.7, 15.96 mg/L O_2_, and 6.5%0 salinity. The bottom water overlying the sediment (measured ~5 cm above the sediment) had a temperature of 2.2°C, pH 7.3, 3.83 mg/L O_2_, and 6.5%0 salinity.

**Figure 1:**
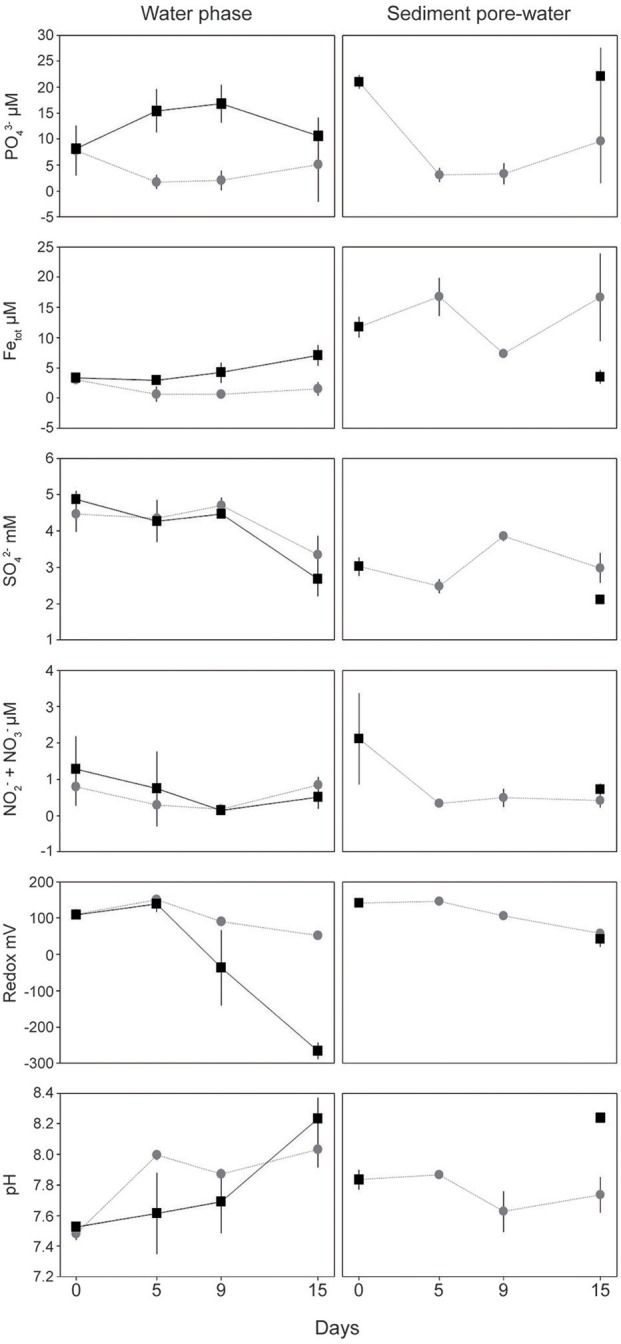
Chemical data from the field (sediment day 0) and laboratory incubations. Water phase and top 1 cm sediment pore-water data are shown on the left and right side, respectively. Gray circles denote the “turned oxic” treatment while black squares denote “turned anoxic.” All values are averages of biological replicates ± 1 SD. Replicates for the “turned oxic” water phase are as follows: day 0 and 5 (*n* = 10), day 9 (*n* = 7), and day 15 (*n* = 4) while the “turned anoxic” were *n* = 3 for all samples except day 15 that was *n* = 2. Replicates from the sediment surface are all averages of triplicates except “turned oxic” day 15 (*n* = 4) and “turned anoxic” were sliced at the end of the incubation after 15 days.

In the water phase of the “turned oxic” treatment, dissolved ${\text{PO}}_{4}^{3-}$ was initially 7.76 ± 4.74 μM on day 0 (number of individual cores (*n*) = 10) and remained <10 μM throughout the experiment with no difference between the days (*n* = 10, 7, and 4 for day 5, 9 and 15, respectively; One-Way ANOVA with *post-hoc* Tukey test, *p* < 0.05; Figure 1). A statistically significantly lower concentration of ${\text{PO}}_{4}^{3-}$ was observed in the “turned oxic” compared to “turned anoxic” water phase at day 15 (*p* < 0.01). Total iron in the “turned oxic” water was initially 3.03 ± 0.69 μM and decreased to <2 μM throughout the experiment (*p* < 0.05 for day 5 and 9; Figure 1). Dissolved ${\text{SO}}_{4}^{2-}$ concentrations in the water phase of “turned oxic” and “turned anoxic” treatments remained stable at 4.51 ± 0.46 mM in the water phase until day 15 at which the concentration decreased to 3.34 ± 0.52 μM and 2.68 ± 0.48 mM, respectively (in the water phase for both treatments; *p* < 0.05). Redox potential in the water phase decreased significantly throughout the experiment in both the “turned oxic” (*p* < 0.05) and “turned anoxic” (*p* < 0.01) treatments. After 15 days of incubation the redox was significantly higher in the “turned oxic” compared to the “turned anoxic” water phase (*p* < 0.01). pH in the water phase increased in both treatments, from 7.48 ± 0.04 to 8.03 ± 0.12 after 15 days in the “turned oxic” (*p* < 0.01), while the “turned anoxic” increased from pH 7.53 ± 0.03 to 8.23 ± 0.14 (*p* < 0.01; Figure 1). Interestingly, measurements of the inorganic sulfur compounds (ISCs) tetrathionate and thiosulfate were stable in the water phase except in the “turned anoxic” day 15 cores in which tetrathionate was higher compared to all other treatments on all days (60.4 ± 0.9 μM compared to <26.8 μM in all other cores; *p* < 0.01). The “turned anoxic” water phase showed similar results, except on day 5 for thiosulfate (146.3 ± 33.3 μM compared to <20.2 μM, *p* < 0.05; one “turned oxic” day 15 outlier removed at 150.3 μM, Supplemental Table 2).

The oxygen penetration depth in the sediment “turned oxic” was 1.69 ± 0.72 mm (*n* = 3), 0.88 ± 0.37 mm (*n* = 3), and 1.24 ± 0.27 mm (*n* = 4) for days 5, 9, and 15, respectively. While the oxygen penetration depth in the sediment “turned anoxic” was 0.00 mm (*n* = 3) when measured on day 15. A full list of all chemistry measurements in the sediment pore-water at the start of the experiment and for each sub-sampling can be found in Supplemental Table 2. In the top 1 cm sediment pore-water collected in the field, dissolved ${\text{PO}}_{4}^{3-}$ was initially 21.02 ± 1.30 μM (*n* = 3) and decreased in the “turned oxic” treatment to 3.12 ± 1.34 and 3.31 ± 2.04 μM on day 5 and 9 of the incubation (*p* < 0.05), respectively. In addition, a significantly lower concentration of ${\text{PO}}_{4}^{3-}$ was observed in the “turned oxic” compared to the “turned anoxic” sediment on day 15 (*p* < 0.05). Dissolved total iron concentrations in the sediment pore-water were initially 11.76 ± 1.69 μM in the field and decreased on day 9 to 7.32 ± 0.65 μM (*p* < 0.05). The sediment cores “turned anoxic” had an iron concentration of 3.52 ± 1.10 μM on day 15 that was statistically significantly decreased compared to the field (*p* < 0.01; Figure 1). Also, the total iron concentration on day 15 was higher in the “turned oxic” sediment compared to the “turned anoxic” with a statistical significance (*p* < 0.01). Dissolved ${\text{SO}}_{4}^{2-}$ concentrations were 3.02 ± 0.24 mM in the field and for the “turned oxic” sediment cores increased significantly on day 9 (*p* < 0.05). The “turned anoxic” sediment pore-water had a ${\text{SO}}_{4}^{2-}$ concentration of 2.11 ± 0.10 mM at the end of the experiment and was significantly lower compared to “turned oxic” sediment at day 9 and 15 (*p* < 0.05; Figure 1). Dissolved ${\text{NO}}_{2}^{-}+$${\text{NO}}_{3}^{-}$ concentrations in the sediment pore-water had an initial concentration of 2.12 ± 1.25 μM in the field compared to <1 μM throughout the incubation for both treatments (*p* < 0.05; Figure 1). The redox potential in the sediment pore-water was initially 141.7 ± 2.2 mV and decreased in both treatments. The sediment pore-water pH in the “turned anoxic” cores increased significantly reaching 8.24 ± 0.03 on day 15 (*p* < 0.05; Figure 1). pH was significantly lower in the “turned oxic” compared to the “turned anoxic” sediment on day 15 (*p* < 0.01). Sediment pore-water tetrathionate concentrations remained stable for both treatments while thiosulfate was only measureable in the “turned oxic” day 15 and “turned anoxic” day 15 sediments (22.5 ± 32.0 μM and 21.3 ± 16.2 μM respectively; Supplemental Table 2).

### Identification of 16S rRNA gene microbial communities

All water and sediment data of identified 16S rRNA gene OTUs are available in Supplemental Data 1. The PMA treatment to distinguish total and viable cells showed no major differences in terms of community structure when tested for statistical significance on phylum, *Proteobacteria* class level, as well as on OTU level (based on One-Way ANOVA statistical testing). Rarefying of OTU counts to the lowest sample size showed no difference in abundant OTU groups (Supplemental Figure 1) and stacked bar graphs of both the total and viable populations are available in Supplemental Figure 2. Rarefaction curves showed that a large portion of the diversity of the bacterial assemblages had been sequenced, with a tendency to a smaller but unknown portion still remaining to be found (Supplemental Figure 3). Therefore, non-rarefied relative abundance values (i.e., OTU counts divided by total sample counts) of cells with an intact membrane (defined as “viable”) are reported hereafter.

**Figure 2:**
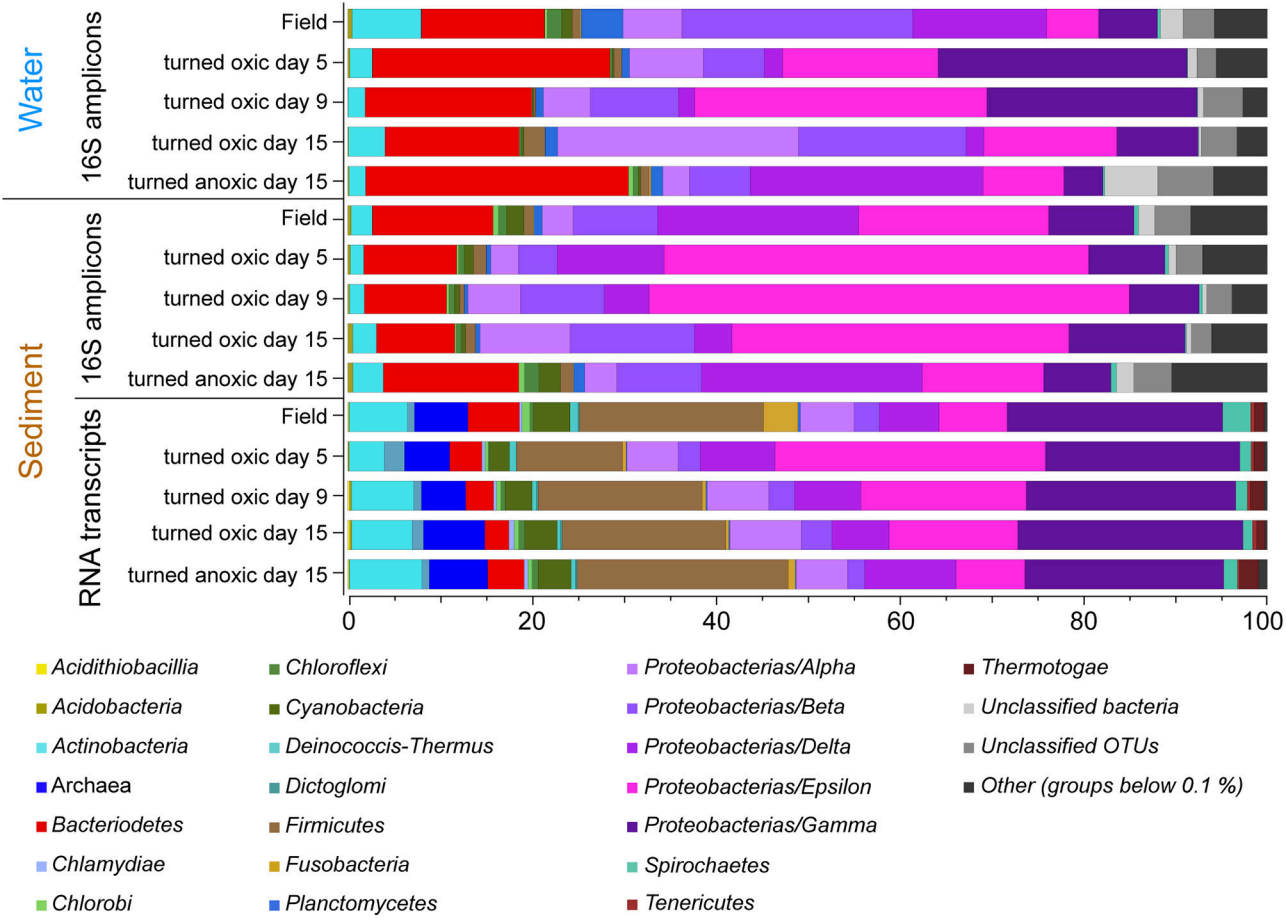
Stacked bar graphs of partial 16S rRNA gene sequences from the water phase and the sediment surface (top 1 cm) collected in the field and throughout the incubation experiment. Averages of relative abundances are shown (*n* = 3, except “turned oxic” water day 9, *n* = 2; and “turned oxic” water and sediment day 15, *n* = 4). The bottom stacked bars show the microbial community derived from UniProtKB/Swiss-Prot reference organisms linked to annotated genes derived from the RNA transcripts (relative proportion of FPKM). *Proteobacteria* have been divided into classes and low abundant groups (< average 0.1% for all samples have been grouped as “Others”).

**Figure 3:**
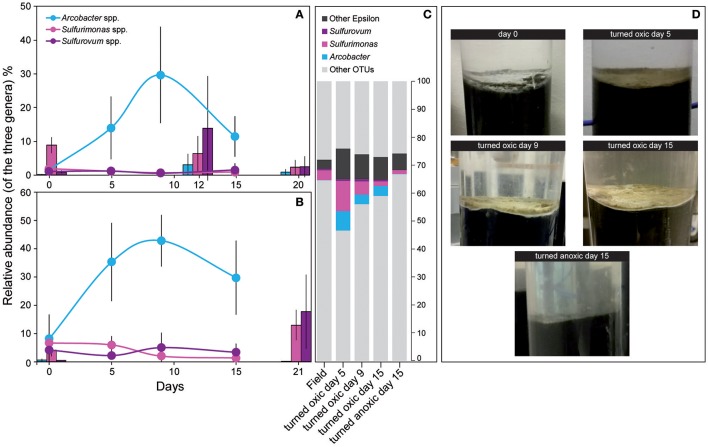
Relative abundance of the three dominant *Epsilonproteobacteri*a genera from the field (day 0) and throughout the incubation experiment. **(A)** Shows 16S rRNA gene relative abundance in the water phase and **(B)** the top 1 cm sediment surface. **(C)** Shows the proportion (% FPKM) of the dominant *Epsilonproteobacteria* derived from metatranscriptomic reads assembled into RNA transcripts and annotated to genes linked to UniProtKB/Swiss-Prot reference organisms (proportion of FPKM). All samples consisted of biological triplicates (*SD* = 1) except sediment and water “turned oxic” day 15 (*n* = 4) and water “turned oxic” day 9 (*n* = 2). The gray areas denote other OTUs. **(D)** Shows photographs of sediment cores throughout the experiment for the “turned oxic” and “turned anoxic” treatments.

Calculations of Shannon H's alpha diversity index showed no differences between treatments or over time in the water phase (Supplemental Table 3). Nevertheless, major changes in water phase taxa were observed as increases in *Alpha-, Epsilon-*, and *Gammaproteobacteria* in cores “turned oxic” (Figure 2). In more detail, *Alphaproteobacteria* in the water of the “turned oxic” cores increased from 6.4 ± 3.9% in the field (*n* = 3) to 26.1 ± 7.8% after 15 days of incubation (*n* = 4; *p* < 0.05, *F* = 15.42, One-Way ANOVA test; SD = 1; Figure 2 and Supplemental Data 1). *Deltaproteobacteria* acted differently depending on if the cores had been turned oxic or anoxic, with a decrease from 14.4 ± 8.4 to 2.0 ± 1.1% in “turned oxic” cores (*p* < 0.05, *F* = 9.27) and were stable at ~25% in the “turned anoxic” cores (Figure 2). Investigating the top 100 abundant OTUs in the water phase, the *Epsilonproteobacteria* were dominated by 16S rRNA gene OTUs aligning with *Arcobacter* spp. while *Rhodobacteraceae* spp. and *Shewanella* spp. were among the top abundant OTUs in the *Alpha-* and *Gammaproteobacteria*, respectively. The sulfate reducing genus *Desulfatiglans* belonging to the *Deltaproteobacteria* was one of the highest abundant OTUs in the water of “turned anoxic” cores (Supplemental Figure 4). The relative abundance of the top abundant *Epsilonproteobacteria* genera (*Arcobacter* spp., *Sulfurimonas* spp., and *Sulfurovum* spp.) was compared to a previous study with a similar experimental setup (Broman et al., 2017a; Figure 3). In the water phase of the “turned oxic” cores, *Arcobacter* spp. had the highest relative abundance and were found to peak during the middle of the incubation experiment and then decline [day 0, 1.6 ± 1.2%; day 5, 14 ± 9.3%; and day 9, 29.7 ± 14.3% (*p* < 0.05, *F* = 13.73), and day 15, 11.5 ± 6.0% (*p* < 0.05, *F* = 7.45)]. In contrast, in the study by Broman et al. (2017a) *Sulfurovum* spp. had a higher abundance and peaked on day 12 during the middle of the experiment (*Acrobacter* spp. ~3%; *Sulfurovum* spp. ~14%; Figure 3). However, there was a trend in the water phase from both experiments that *Arcobacter* spp. increased and then started to decline at some point during the incubation (Figure 3).

**Figure 4:**
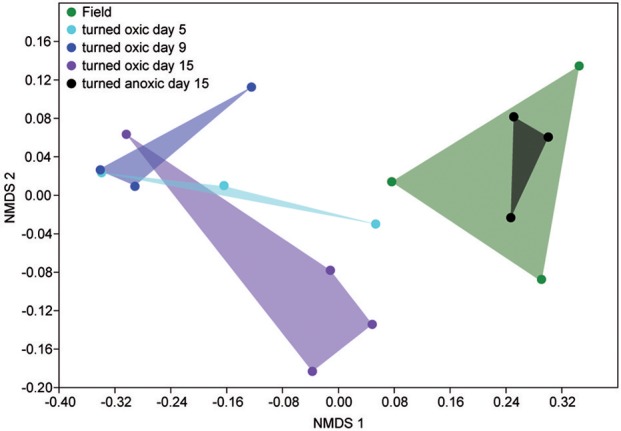
NMDS plot based on the relative abundance of sediment 16S rRNA gene OTUs. Shaded areas denote: field samples, green; “turned oxic” day 5, turquoise; “turned oxic” day 9, blue; “turned oxic” day 15, purple; “turned anoxic” day 15, black.

**Figure 5:**
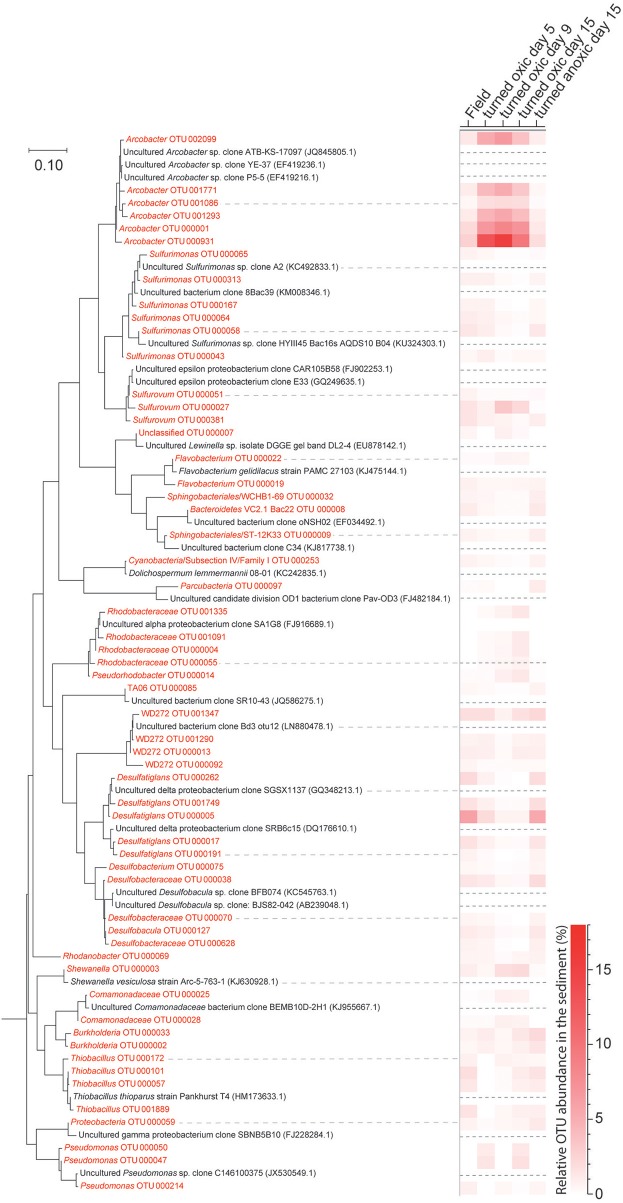
Phylogenetic maximum likelihood tree and the heatmap showing OTUs derived from the 16S rRNA gene analysis from collected sediment (top 1 cm) with a total relative abundance higher than 0.3% (average of all samples). Sequences from this study are colored red while reference sequences downloaded from NCBI Genebank are colored black and have been marked with a dashed line in the heatmap. A marine *Actinomycete* (NCBI accession: AJ866956.1) was used to root the tree (not shown) and the tree with the highest log likelihood (−7167.1443) is presented. The scale bar represents nucleotide substitutions per site.

In the top 1 cm of the sediment surface, alpha diversity (Shannon's H index) decreased significantly in the cores “turned oxic” on day 9 compared to the sediment in the field (6.42 ± 0.31, 7.56 ± 0.08, respectively; *F* = 39.0, *p* < 0.01). Also, alpha diversity at the end of the experiment in the sediment of the “turned oxic” cores compared to the “turned anoxic” cores was statistically different (6.81 ± 0.56 and 7.81 ± 0.21, respectively; *F* = 8.28, *p* < 0.05; Supplemental Table 3). Beta diversity of the microbial community structure in the “turned oxic” sediment increased throughout the experiment compared to the field samples, with “turned oxic” day 5 (Bray-Curtis dissimilarity index 0.58; with values of 0 indicating a similar community structure and 1 high dissimilarity; Supplemental Data 2), day 9 (0.65), and day 15 (0.70). In addition, beta diversity of the sediment community showed a high dissimilarity between the “turned oxic” and “turned anoxic” sediment at day 15 (0.72). Multivariate NMDS analysis also showed dissimilarities in the microbial community structure between the “turned oxic” and the other samples (i.e., field and “turned anoxic”; Figure 4). Major changes in sediment taxa were related to an increase of *Epsilonproteobacteria* and a decrease of *Deltaproteobacteria* and the phylum *Bacteroidetes* in the “turned oxic” cores (Figure 2). Of these, the largest changes observed in the top 1 cm sediment surface were related to the *Epsilonproteobacteria* which increased from 20.0 ± 9.6% in the field (*n* = 3) to 45.0 ± 11.1% on day 5 in the “turned oxic” cores (*n* = 3; *p* < 0.05, *F* = 8.68) and 50.9 ± 3.9% on day 9 (*n* = 3; *p* < 0.01, *F* = 26.58) before decreasing again to 35.4 ± 16.6% on day 15 (*n* = 4, non-significant; Figure 2 and Supplemental Data 1). In the same cores, *Deltaproteobacteria* in the sediment declined sharply from 21.4 ± 12.2 to 4.3 ± 1.1% on day 15 (*p* < 0.05, *F* = 8.25). Among the most abundant OTUs, the majority of the *Epsilonproteobacteria* 16S rRNA gene sequences were affiliated with *Arcobacter* spp. (~75% of all *Epsilonproteobacteria* in the sediment and up to 43% of the whole community), as well as a lower relative abundance of *Sulfurimonas* spp. and *Sulfurovum* spp. (<13% of all sediment *Epsilonproteobacteria* and < 4% of the whole community; Figures 3, 4 and Supplemental Figure 5). Dominating OTUs in the *Deltaproteobacteria* class belonged to the sulfate reducing genus *Desulfatiglans* (~45% of all sediment *Deltaproteobacteria*; Figure 5 and Supplemental Figure 5). However, these OTUs were not affiliated with organisms in the NCBI GenBank taxonomy database (Figure 5). In the sediment surface, the relative abundance of *Arcobacter* spp. increased throughout the experiment (*p* < 0.01, *F* = 23.00) and then declined again on day 15 (Figure 3). As sediment was only sliced on days 0 and 21 in the study by Broman et al. (2017a) the results from the two studies cannot not be directly compared.

### 16S rRNA gene OTUs and chemical correlations

Pearson correlations of the top 20 abundant viable OTUs in the water vs. the sediment showed significant positive correlations of all *Arcobacter* spp. in the water and sediment (*p* < 0.01, *r* > 0.7; Supplemental Data 3). This indicated a concomitant increase in relative abundance of *Arcobacter* spp. in both the sediment surface and the water phase. In the same correlation matrix, *Desulfatiglans* spp. negatively correlated to many of the *Arcobacter* spp. OTUs (*p* < 0.05, *r* < −0.5) as well as strongly negatively correlating to oxygen concentrations in the water phase and thus, favoring anoxic conditions (*p* < 0.01, *r* < −0.9; Supplemental Table 4). From the correlation network of sediment 16S rRNA gene OTUs, statistically significant interactions with *Arcobacter* OTUs were extracted (*r* < −0.7 or > 0.7, and *p* < 0.01). These positively correlated with OTUs belonging to *Shewanella, Sulfurovum*, and *Sulfurospirillum* and negatively with *Desulfatiglans* OTUs (Supplemental Figure 6).

16S rRNA gene OTUs were divided into taxonomical groups (phylum, class, and genera) and correlated against the measured chemical parameters. In the water phase, total iron and redox potential had significant correlations with *Deltaproteobacteria*. The abundant genus *Desulfatiglans* was found to positively correlate with total iron (*p* < 0.01, *r* > 0.7) and negatively with redox potential (*p* < 0.01, *r* < −0.8), which suggested *Desulfatiglans* spp. favored an anoxic environment (Supplemental Table 5).

In the sediment the highly abundant *Deltaproteobacteria* positively correlated with pH and ${\text{PO}}_{4}^{3-}$ (*p* < 0.05, *r* > 0.5) while the abundant *Epsilonproteobacteria* negatively correlated to both of these parameters (*p* < 0.01, *r* < -0.5; Supplemental Table 5). Similar to *Deltaproteobacteria*, the dominant genus *Desulfatiglans* within this class positively correlated with pH and ${\text{PO}}_{4}^{3-}$ (*p* < 0.05, *r* > 0.5). In contrast, the highly abundant genus arcobacter belonging to the class *Epsilonproteobacteria* correlated negatively to both pH and ${\text{PO}}_{4}^{3-}$ (*p* < 0.05, r < −0.5; Supplemental Table 5).

### Metatranscriptomics of community structure and metabolic functions in the sediments

The *de novo* assemblies resulted in 431522 RNA transcripts. On average 20 ± 5% were annotated and the remaining RNA transcripts had no hit in the UniProtKB/Swiss-Prot database. The annotated transcripts resulted in 19074 unique genes corresponding to an average of 4,786 ± 1,627 genes per sample (*n* = 17). A full list of annotated RNA transcripts with TMM normalized FPKM values is available in Supplemental Data 4.

Microbial community activity derived from the number of RNA transcript reads annotated to genes linked to UniProtKB/Swiss-Prot reference organisms also showed a large abundance of *Epsilonproteobacteria* in the “turned oxic” sediment. The *Epsilonproteobacteria* proportion of the FPKM was ~7% in the field compared to ~29, 18, and 14% on days 5, 9, and 15, respectively; Figure 2). In contrast, and as observed in the field, the *Epsilonproteobacteria* in the “turned anoxic” sediment on day 15 accounted for ~7% of annotated RNA transcripts. Differences in the RNA transcript data compared to the 16S rRNA gene amplicons included a larger proportion of Archaea (~6%), *Firmicutes* (~18%), and *Gammaproteobacteria* (~23%) which were present throughout the experiment in all treatments (Figure 2). In addition, activity of *Arcobacter* and *Sulfurimonas* was also observed in the metatranscriptome which resulted in an increase from ~0.1% in the field (proportion of FPKM) to ~7, ~4, and ~4% on days 5, 9, and 15, respectively. Similar to *Arcobacter*, the genus *Sulfurimonas* was assigned a substantial part of annotated transcripts and accounted for ~3% in the field and ~11, ~5, and ~2% on day 5, 9, and 15, respectively (Figure 3). *Sulfurimonas* spp. and *Sulfurovum* spp. decreased throughout the incubation experiment in the sediment surface, from a relative abundance of ~7 and ~1% to ~1 and 0.5%, respectively (Figure 3).

Due to the high variation among the sediment cores, differential expression analysis between the treatments using edgeR resulted in high false discovery rate values (>0.05 FDR), and therefore, statistical analysis was not reliable. Instead, we decided to investigate what genes were present with a FPKM value higher than 1,000 (for at least one of the samples) and that could be linked to the Archaea and Bacteria kingdoms. This resulted in a datasheet of 1,103 genes that were categorized into e.g., sulfur, nitrogen, and methane metabolism using the Kegg, MetaCyc, and GO biological terminology databases. In addition, the portion of genes that could directly be linked to the genus *Arcobacter* was extracted.

**Figure 6:**
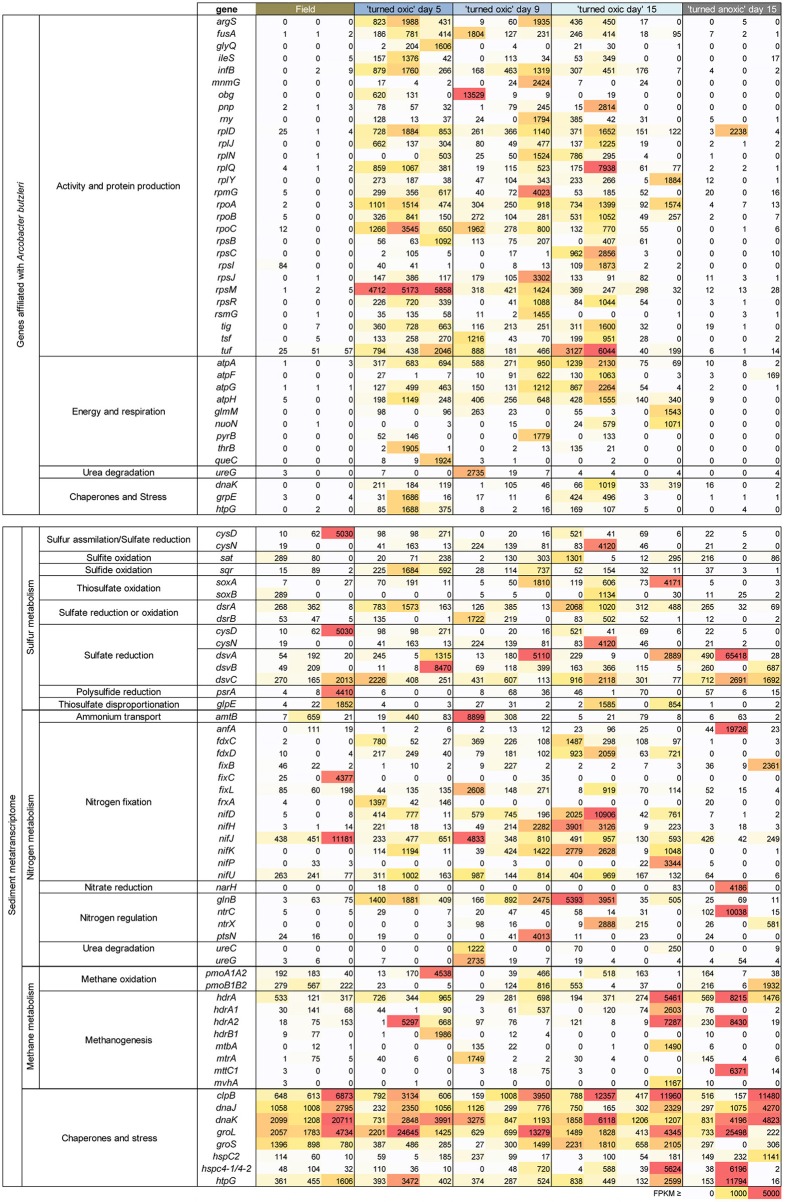
Heatmap showing archaea and bacteria genes with more than 1,000 FPKM in at least one sample derived from the metatranscriptomes. The genes shown were chosen based on all genes that could directly be linked to an isolate of *Arcobacter* available in the UniProtKB/Swiss-Prot database (upper box), and/or chemistry processes in the sediments (lower box). Replicates (individual sediment cores) are represented as individual columns below each treatment. The color gradient denotes the level of FPKM counts with 0–1,000 as a white-yellow gradient, 1,001–4,999 as a yellow-orange-red gradient, and ≥ 5,000 FPKM as red.

The portion of genes directly annotated to the genus *Arcobacter* (closest species in UniProtKB/Swiss-Prot database was *Arcobacter butzleri*) were mainly related to ribosomal activity/protein synthesis and energy and respiration (Figure 6). In more detail, the majority of genes for ribosomal (e.g., *rpl*D, *rplJ*, and *rplN*), RNA polymerase (e.g., *rpoA*), and tRNA activities (*argS, ileS*, and *mnmG*) were < 85 FPKM in the field and >1,000 FPKM throughout most “turned oxic” sediment incubation replicates (Figure 6). Genes involved in energy and respiration such as ATP synthase (e.g., *atpA* and *atpF*) and NADH-quinone oxidoreductase (i.e., aerobic respiration, *nuoN*) were also higher compared to the field (Figure 6).

**Figure 7:**
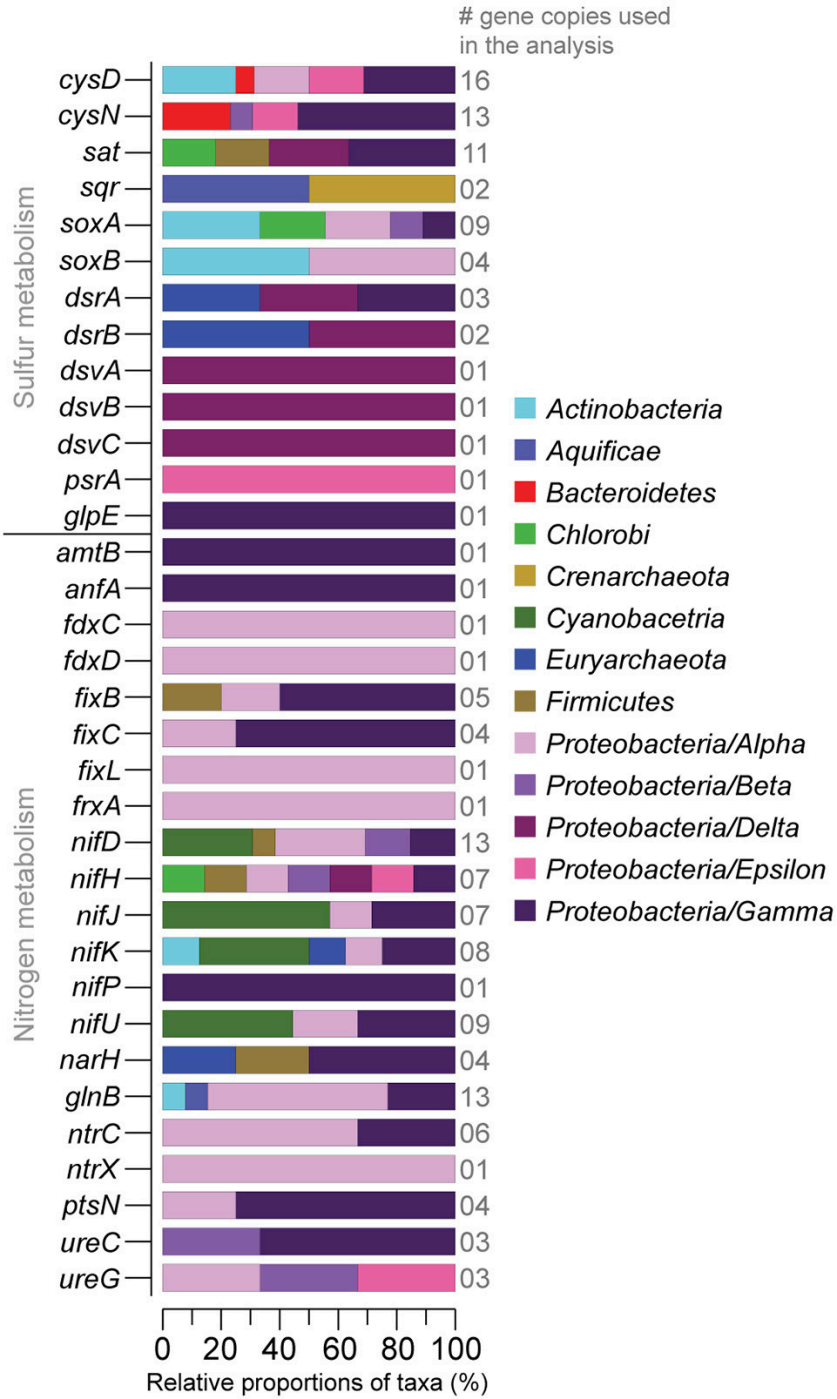
Relative proportions of taxa (%) sharing sulfur and nitrogen related genes derived from the sediment metatranscriptomes (>1,000 FPKM in at least one sample). The number of gene copies used in the taxonomic affiliation analysis is shown on the right-side y-axis. The stacked bars show the taxonomic affiliation of the genes in the UniProtKB/Swiss-Prot database. Proteobacteria have been divided into classes.

RNA transcripts ascribed to the genes *cysD* and *cysN* (heteromeric sulfate adenylyltransferase) involved in e.g., sulfur assimilation (Fera et al., 2004) showed a variance with one field core having a *cysD* FPKM of 5,030 and one “turned oxic” day 15 core having a *cysN* FPKM of 4120. The gene *sat* encoding homomeric sulfate adenylyltransferase but used in oxidation of sulfite to sulfate was present in the field samples and throughout the incubation experiment. RNA transcripts ascribed to *sqr* (sulfide:quinone oxidoreductase) involved in sulfide oxidation to sulfur were present in the field and also throughout the incubation. RNA transcripts encoding thiosulfate oxidation protein (*soxAB*) had a tendency to increase from 0 to 298 FPKM in the field to 73-4171 FPKM in the “turned oxic” day 15 sediment cores (Figure 6). In contrast, RNA transcripts ascribed to *soxAB* were lower in the “turned anoxic” sediment (≤26 FPKM). The genes *dsrA* and *dsrB* (dissimilatory sulfite reductase) used in sulfate reduction or in reverse for sulfur oxidation (Harada et al., 2009) were <370 FPKM in the field and >1,000 FPKM in several “turned oxic” cores throughout the experiment (Figure 6). Also used in sulfate reduction, the genes *dsvA, dsvB*, and *dsvC* (dissimilatory sulfite reductase) were present in the field and throughout the oxygenation experiment with a tendency of lower values in the “turned anoxic” cores (with “turned oxic” day 15, 312-42068 FPKM; and “turned anoxic” day 15, 32-265 FPKM). However, in contrast to *dsrAB*, the *dsvABC* genes were higher in the “turned anoxic” sediment (28–65,418 FPKM for *dsvA*, 0–687 FPKM for *dsvB*, and 712–2,961 FPKM for *dsvC*). RNA transcripts ascribed to polysulfide reductase (*psrA*) involved in H_2_S formation occurred mainly in the sediment of one field core with a FPKM value of 4,410 (Figure 6). Several different reference organisms were linked to sulfur cycling genes in the UniProtKB/Swiss-Prot database, including *Actinobacteria, Bacteriodetes, Epsilon-*, and *Gammaproteobacteria* for *CysDN* (Figure 7), and e.g., *Chlorobi, Firmicutes, Delta*-, and *Gammaproteobacteria* for *sat* (Figure 7). In more detail, the *Deltaproteobacteria* family *Desulfobacteraceae* was found to be predominantly affiliated with *sat* (% of FPKM values; Supplemental Data 5). The phyla *Aquificae* and *Crenarchaeota* shared the most *sqr* genes in the metatranscriptome and was dominated by the family *Aquificaceae* (Supplemental Data 5). The *soxAB* genes were shared among a wide variety of taxa including *Actinobacteria* and *Alphaproteobacteria* (Figure 7) and upon closer inspection, *soxA* was dominated by the *Chlorobiaceae* and *soxB* was shared between the *Corynebacteriaceae, Rhodobacteraceae*, and *Rhizobiaceae* families (Supplemental Data 5). The *Chromatiaceae* dominated *dsrA*, while the *Archaeoglobaceae* and *Desulfovibrionaceae* families shared genes for *dsrB* (Figure 6 and Supplemental Data 5). The *dsvABC* genes were dominated by the *Desulfovibrionaceae* (Figure 7 and Supplemental Data 5).

RNA transcripts attributed to nitrogen fixation proteins (e.g., *fdxCD, fixL, frxA*, and *nifDHJKP*) were predominantly higher (notable exceptions being *fixC*, and *nifJ*) in the “turned oxic” sediment throughout the experiment (Figure 6). In contrast, only the *anfA* and *fixB* nitrogen fixation proteins were higher in the “turned anoxic” cores compared to “turned oxic” day 15 (Figure 6). Several genes involved in nitrogen regulation (e.g., *glnB, nrtX*, and *ptsN*) were ≤75 FPKM in the field and >1,000 FPKM in several of the “turned oxic” cores (Figure 6). Similar to the sulfur cycling genes, a wide variety of taxa were involved in nitrogen cycling, such as *Alphaproteobacteria* (in the delimited >1000 FPKM dataset; Figure 7). In more detail, *fdxD* was affiliated with the *Rhodobacteraceae* (Supplemental Data 5). The *fixL* gene was distributed among the *Alphaproteobacteria* families *Xanthobacteraceae, Bradyrhizobiaceae*, and *Rhizobiaceae* while *frxA* was only affiliated with *Bradyrhizobiaceae* (Figure 7 and Supplemental Data 5). The *nifDHJKPU* genes were distributed among a wide variety of taxa including, e.g., *Cyanobacteria*, and *Alpha*-, *Beta*-, *Delta*-, *Epsilon*-, and *Gammaproteobacteria* (Figure 7). Upon closer inspection of the unevenly shared *nifDHJKPU* genes, the *Helicobacteraceae* dominated the *nifH* gene and *Nostocaceae* dominated *nifU* (Supplemental Data 5). The genes *nifP* and *anfA* were only affiliated with the *Pseudomonadaceae* (Figure 7 and Supplemental Data 5). Most of the *glnB* and *nrtCX* were affiliated with *Alphaproteobacteria* (Figure 7). Finally, *ureC* and *ureG* genes were distributed among *Alpha*-, *Beta*-, and *Gammaproteobacteria* (Figure 7) with the *Campylobacteraceae* dominating *ureG* in the “turned oxic” cores and the *Methylobacteriaceae* only being present in the field and “turned anoxic” cores (Supplemental Data 5).

RNA transcripts attributed to genes involved in methane metabolism varied throughout the experiment, with *pmoA1A2* being present in the field and throughout the incubation experiment (Figure 6). Genes for methanogenesis included the heterodisulfide reductase subunits A1 and A2 (*hdrA1* and *hdrA2*, respectively) appearing in both the “turned oxic” and “turned anoxic” sediments (Figure 6). Other genes involved in methanogenesis from e.g., methylamine and CO_2_ were also present in the field and throughout the incubation experiment such as *mtbA, mtrA*, and *mvhA* (Figure 6). The taxonomical annotation of genes related to methane metabolism in the UniProtKB/Swiss-Prot database was diverse and included the archaeal phylum *Euryarchaeota* (*hdrA1, hdrA2, hdrB1, mtbA, mtrA, mttC1*, and *mvhA*), the bacterial phylum *Actinobacteria* (*mtrA*), and the *Gammaproteobacteria* class (*pmoA1A2* and *pmoB1B2*) (Supplemental Data 4).

RNA transcripts attributed to the genes coding for the chaperones *dnaJ* and *dnaK* were present in all treatments (with values >1,000 FPKM in most replicates; Figure 6). Similar results were found for other chaperones, e.g., *groL* and *htpG* (Figure 6). Heat-shock proteins varied in the treatments with, e.g., *hspC* having <1,000 FPKM except in one “turned anoxic” core (1141 FPKM). A wide variety of reference organisms in the UniProtKB/Swiss-Prot database were linked to these stress related genes, including the archaeal phylum *Euryarchaeota* and several bacterial phyla, e.g., *Actinobacteria, Bacteroidetes, Tenericutes, Verrucomicrobia*, and *Firmicutes* plus the *Alpha-, Beta-, Delta-, Epsilon-*, and *Gammaproteobacteria* classes (Supplemental Data 4).

## Discussion

Dissolved oxygenation concentrations in the oceans have decreased by ~2% since the 1960s and are suggested to further decrease due to increased global seawater warming (Schmidtko et al., 2017). In addition, severe hypoxia has developed in the Baltic Sea during the last century due to anthropogenic eutrophication (Conley et al., 2002; Carstensen et al., 2014). Re-oxygenation of anoxic bottom zones by pumping oxygen rich surface water decreases phosphate and H_2_S from the sediment (Stigebrandt et al., 2015) as well as increasing the abundance of microorganisms and RNA transcripts related to sulfur cycling (Broman et al., 2017a). Here we further increase knowledge of hypoxic bottom zones by following changes shortly after oxygenation of oxygen deficient sediment using chemistry measurements, 16S rRNA gene amplicons, and metatranscriptomics to infer the microbial community and its active metabolic processes.

Throughout the experiment, the most significant changes in water and sediment chemistry occurred on days 5 and 9 (Figure 1). This was also the time period when the relative abundance and activity of *Arcobacter* spp. reached a peak before declining on day 15 (Figures 2, 3). Both cultivable (*Arcobacter butzleri*) and uncultured *Arcobacter* spp. are found in marine coastal waters (Fera et al., 2004) and Candidatus *Arcobacter sulfidicus* is highly competitive in the anoxic-oxic interface as it can withstand high concentrations of H_2_S and very low concentrations of O_2_ (Sievert et al., 2007). Candidatus *A. sulfidicus* produces a white, filamentous sulfur film (Wirsen et al., 2002) that is derived from the incomplete oxidation of sulfide (Sievert et al., 2007). The genus *Arcobacter* is metabolically versatile and has for example been observed to reduce manganese oxides heterotrophically in the oxic-anoxic interface and sulfidic anoxic water (Berg et al., 2013) as well as anoxic manganese-oxide rich sediment (Vandieken et al., 2012; Berg et al., 2013). However, *Arcobacter* genes attributed to manganese reduction could not be identified in the dataset. Although sulfide, sulfur, and thiosulfate oxidation was indicated in the “turned oxic” sediment metatranscriptome throughout the experiment, these genes could not be directly attributed to, e.g., *Arcobacter butzleri*. However, the potential importance of *Arcobacter* spp. was indicated by high RNA transcript counts for genes encoding ribosomal activity/protein synthesis along with energy and respiration (e.g., ATP synthase and NADH-quinone oxidoreductase; Figure 6). Here it was shown that re-oxygenation of oxygen deficient coastal sediment triggered the growth of *Arcobacter* spp. in just 5 days, suggesting they are key players in the microbial community and influence cycling of chemical substrates.

Alongside the increase of *Arcobacter* OTUs and their RNA transcripts, a pronounced increase in RNA transcripts attributed to *Sulfurimonas* spp. was detected despite that OTUs attributed to this genus were not in high abundance in the 16S rRNA gene data (Figure 3). The genus *Sulfurimonas* has previously been reported in sediments and encompasses microaerophilic and facultative anaerobic species able to oxidize sulfide/zero-valent sulfur and reduce nitrate (Han and Perner, 2015). RNA transcripts for sulfur oxidation genes by *Sulfurimonas* were not detected in the RNA data, but populations from this genus were active based on ribosomal and RNA polymerase transcripts (Supplemental Data 4). In a previous study oxygenating anoxic sediment, the genera *Sulfurimonas* and *Sulfurovum* were shown to be favored upon oxygenation due to their increase in relative abundance after 21 days of oxygenation (Broman et al., 2017a). Throughout the experiment genes for sulfur cycling, such as sulfide oxidation, were maintained during the incubation and affiliated with a large variety of taxa indicating active sulfur cycling during oxygenation. Taken together, these findings suggest that *Arcobacter* and *Sulfurimonas* were active upon re-oxygenation and sulfur cycling was maintained by a variety of microorganisms in the sediment. However, it was possible that the abundant *Epsilonproteobacteria* in these systems could be indirectly linked to sulfur cycling by microbial symbiosis or directly through unknown metabolic pathways.

In addition to results related to sulfur cycling, RNA transcripts for N_2_-fixation were detected in the field and maintained in “turned oxic” sediment (Figure 6). N_2_-fixation in oxic environments has previously been described from rate measurements in oligotrophic marine waters (Rahav et al., 2013) and a contributing factor to N_2_-fixation in the sediment could therefore be the low concentration of available ${\text{NO}}_{3}^{-}$ in the pore-water (<2 μM; Figure 1). Recent data indicate that that the prevalence of N_2_-fixation in sediments has been underestimated, including nitrogen rich sediments and N_2_-fixation in conjunction with denitrification (Newell et al., 2016). It is therefore possible that the incubation of sediment in darkness contributed to triggering the N_2_-fixation genes, i.e., when photosynthetic production of oxygen does not interfere with N_2_-fixation (Toepel et al., 2008). Furthermore, the RNA transcript data indicated that a wide variety of taxonomical families belonging predominantly to *Alphaproteobacteria* but also other phyla such as *Cyanobacteria, Epsilon*-, and *Gammaproteobacteria* conducted N_2_-fixation (Figure 7). In relation to the *Arcobacter* spp. observed in this study, *Arcobacter nitrofigilis* (McClung and Patriquin, 1980; Pati et al., 2010) and the filamentous sulfur producing *Arcobacter sulfidicus* also fix N_2_ (Wirsen et al., 2002). It is therefore possible that the uncultured *Arcobacter* spp. produced filamentous sulfur in conjunction with nitrogen fixation under microaerophilic conditions. However, future additions to the reference databases will allow firm conclusions regarding the microorganisms responsible for N_2_-fixation. The community RNA expression analysis indicated that the high heterogeneity in sediments creates microniches that permit anaerobic N_2_-fixation during early stage re-oxygenation and that this process is underestimated under these conditions.

It is relevant to consider that the effect of turbidity on the effectiveness of PMA to measure cell viability has been discussed (e.g., Desneux et al., 2015) and total DNA and PMA-treated microbial communities in water were found to be similar upon sequencing. Therefore, RNA-seq is suggested as a better alternative (Li et al., 2017). As we could not detect any differences between the viable and total communities, we conclude that the abundant taxonomical groups were principally viable (and this was further verified with the RNA data). However, differences were observed in relative proportion of taxa between the 16S rRNA gene OTU and metatranscriptomic data. Possible explanations can be primer bias during 16S rRNA gene amplification along with differences in rate of cellular activity and/or chromosome copies (Soppa, 2017). It should also be noted that rarifying the complete viable OTU dataset did not yield differences in the results and conclusions from this study, indicating that the sample size was sufficient to adequately reflect the distribution of populations in our samples.

In conclusion, oxygenation of coastal oxygen deficient sediment caused a change in the relative abundance of *Arcobacter*-like and *Sulfurimonas*-like species in just 5 days. RNA transcripts also indicated that the *Arcobacter*-like species were active in the sediment after oxygenation. In addition, RNA transcripts attributed to N_2_-fixation genes were predominant in the metatranscriptomes in both the field and oxygenated samples. These findings are in accordance to previous observations of N_2_-fixation rates in oxic sediments and confirm the importance of N_2_-fixation upon oxygen shifts. In addition to previous findings that toxic H_2_S is released from the sediment and oxidized (Broman et al., 2017a), we further demonstrate that metatranscriptomic signatures for sulfur cycling were maintained upon oxygenation of the oxygen deficient sediments. Finally we show that organic nitrogen is potentially added to the system by continued N_2_-fixation upon oxygenation. Both of these results would help the re-establishment of macroorganism communities in the re-oxygenated benthic zone.

## Author contributions

EB designed the study, sampled in the field, conducted laboratory work, analyzed data and drafted the manuscript; VS helped during field sampling and assisted in the laboratory; MD and JP designed the study and helped to draft and revise the manuscript. All authors read and approved the final manuscript.

### Conflict of interest statement

The authors declare that the research was conducted in the absence of any commercial or financial relationships that could be construed as a potential conflict of interest.
