# Supplementary material for: Oxygenation of Hypoxic Coastal Baltic Sea Sediments Impacts on Chemistry, Microbial Community Composition, and Metabolism
Source: Front Microbiol. 2017 Dec 12;8:2453. doi: 10.3389/fmicb.2017.02453 (PMC5733055; doi:10.3389/fmicb.2017.02453)
Supplement: Supplementary file 6 [file DataSheet6.pdf]

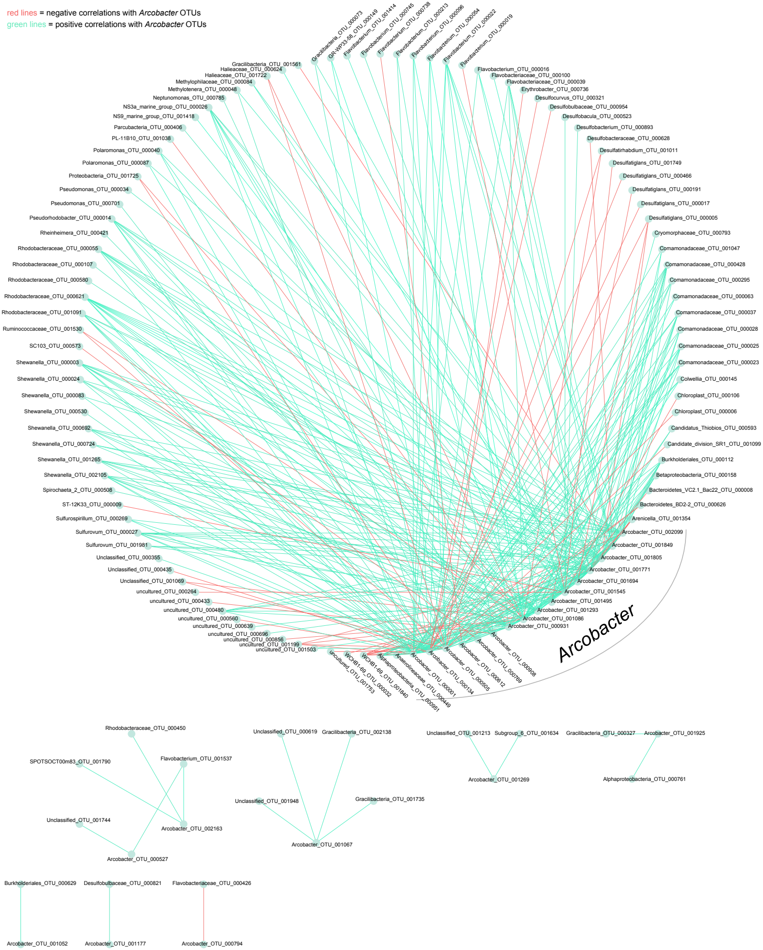

red lines = negative correlations with *Arcobacter* OTUs
green lines = positive correlations with *Arcobacter* OTUs
Gracilibacteria_OTU_000073
GR-WP33-58_OTU_000149
Flavobacterium_OTU_001414
Flavobacterium_OTU_000745
Flavobacterium_OTU_000738
Flavobacterium_OTU_000213
Flavobacterium_OTU_000096
Flavobacterium_OTU_000054
Flavobacterium_OTU_000022
Flavobacterium_OTU_000019
Gracilibacteria_OTU_001561
Halieaceae_OTU_000624
Halieaceae_OTU_001722
Methylophilaceae_OTU_000084
Methylotenera_OTU_000048
Neptunomonas_OTU_000785
NS3a_marine_group_OTU_000026
NS9_marine_group_OTU_001418
Parcubacteria_OTU_000406
PL-11B10_OTU_001038
Polaromonas_OTU_000040
Polaromonas_OTU_000087
Proteobacteria_OTU_001725
Pseudomonas_OTU_000034
Pseudomonas_OTU_000701
Pseudorhodobacter_OTU_000014
Rheinheimera_OTU_000421
Rhodobacteraceae_OTU_000055
Rhodobacteraceae_OTU_000107
Rhodobacteraceae_OTU_000580
Rhodobacteraceae_OTU_000621
Rhodobacteraceae_OTU_001091
Ruminococcaceae_OTU_001530
SC103_OTU_000573
Shewanella_OTU_000003
Shewanella_OTU_000024
Shewanella_OTU_000083
Shewanella_OTU_000530
Shewanella_OTU_000692
Shewanella_OTU_000724
Shewanella_OTU_001265
Shewanella_OTU_002105
Spirochaeta_2_OTU_000508
ST-12K33_OTU_000009
Sulfurospirillum_OTU_000269
Sulfurovum_OTU_000027
Sulfurovum_OTU_001981
Unclassified_OTU_000355
Unclassified_OTU_000435
Unclassified_OTU_001069
uncultured_OTU_000264
uncultured_OTU_000433
uncultured_OTU_000480
uncultured_OTU_000560
uncultured_OTU_000639
uncultured_OTU_000696
uncultured_OTU_000856
uncultured_OTU_001199
uncultured_OTU_001503
uncultured_OTU_001753
WCHB1-69_OTU_000032
WCHB1-69_OTU_001840
Alphaproteobacteria_OTU_000951
Anaerolineaceae_OTU_000449
Arcobacter_OTU_000001
Arcobacter_OTU_000134
Arcobacter_OTU_000505
Arcobacter_OTU_000612
Arcobacter_OTU_000769
Arcobacter_OTU_000908
Flavobacterium_OTU_000016
Flavobacteriaceae_OTU_000100
Flavobacteriaceae_OTU_000039
Erythrobacter_OTU_000736
Desulfocurvus_OTU_000321
Desulfobulbaceae_OTU_000954
Desulfobacula_OTU_000523
Desulfobacterium_OTU_000893
Desulfobacteraceae_OTU_000628
Desulfatirhabdium_OTU_001011
Desulfatiglans_OTU_001749
Desulfatiglans_OTU_000466
Desulfatiglans_OTU_000191
Desulfatiglans_OTU_000017
Desulfatiglans_OTU_000005
Cryomorphaceae_OTU_000793
Comamonadaceae_OTU_001047
Comamonadaceae_OTU_000428
Comamonadaceae_OTU_000295
Comamonadaceae_OTU_000063
Comamonadaceae_OTU_000037
Comamonadaceae_OTU_000028
Comamonadaceae_OTU_000025
Comamonadaceae_OTU_000023
Colwellia_OTU_000145
Chloroplast_OTU_000106
Chloroplast_OTU_000006
Candidatus_Thiobios_OTU_000593
Candidate_division_SR1_OTU_001099
Burkholderiales_OTU_000112
Betaproteobacteria_OTU_000158
Bacteroidetes_VC2.1_Bac22_OTU_000008
Bacteroidetes_BD2-2_OTU_000626
Arenicella_OTU_001354
Arcobacter_OTU_002099
Arcobacter_OTU_001849
Arcobacter_OTU_001805
Arcobacter_OTU_001771
Arcobacter_OTU_001694
Arcobacter_OTU_001545
Arcobacter_OTU_001495
Arcobacter_OTU_001293
Arcobacter_OTU_001086
Arcobacter_OTU_000931
Arcobacter
Rhodobacteraceae_OTU_000450
SPOTSOCT00m83_OTU_001790
Flavobacterium_OTU_001537
Unclassified_OTU_001744
Arcobacter_OTU_002163
Arcobacter_OTU_000527
Unclassified_OTU_000619
Gracilibacteria_OTU_002138
Unclassified_OTU_001948
Gracilibacteria_OTU_001735
Arcobacter_OTU_001067
Unclassified_OTU_001213
Subgroup_6_OTU_001634
Arcobacter_OTU_001269
Gracilibacteria_OTU_000327
Arcobacter_OTU_001925
Alphaproteobacteria_OTU_000761
Burkholderiales_OTU_000629
Desulfobulbaceae_OTU_000821
Flavobacteriaceae_OTU_000426
Arcobacter_OTU_001052
Arcobacter_OTU_001177
Arcobacter_OTU_000794
