# Supplementary material for: Oxygenation of Hypoxic Coastal Baltic Sea Sediments Impacts on Chemistry, Microbial Community Composition, and Metabolism
Source: Front Microbiol. 2017 Dec 12;8:2453. doi: 10.3389/fmicb.2017.02453 (PMC5733055; doi:10.3389/fmicb.2017.02453)
Supplement: Supplementary file 7 [file DataSheet7.docx]

Supplementary Material

Oxygenation of hypoxic coastal Baltic Sea sediments impacts on chemistry, microbial community composition, and metabolism

Elias Broman*, Varvara Sachpazidou, Jarone Pinhassi, Mark Dopson

*** Correspondence:** Elias Broman: elias.broman@lnu.se

**Supplemental Data 1.** Clustered OTUs annotated against the SILVA taxonomy 16S rRNA database. The table consists of total and viable cells from the water phase and sediment collected in the field and throughout the experiment. Biological replicates are indicated by number 1 to 4. A separate Excel file has been uploaded.

**Supplemental Data 2**. The table and insert heatmap shows Bray-Curtis beta diversity for the viable 16S rRNA gene sediment samples (calculated on OTU level), with a value of 1 indicating high dissimilarities among samples. The insert plot shows an NMDS plot based on relative abundance of 16S rRNA gene OTUs constructed in the software past 3.10. A separate Excel file has been uploaded.

**Supplemental Data 3**. Pearson correlations of the top 20 abundant OTUs (viable cells) in the water phase and sediment surface (top 1 cm). A separate Excel file has been uploaded.

**Supplemental Data 4**. The first table shows the proportion of identified rRNA sequences, unclassified transcripts, and the amount of identified genes. The second table shows all UniProtKB/Swiss-Prot hits (gene and protein names), the Gene Ontology functional category ‘biological processes’, EC number, Kegg Orthology (KO) identifier, and the closest taxonomical hit in the UniProtKB/Swiss-Prot database. Read counts are shown as TMM normalized FPKM values. A separate Excel file has been uploaded.

**Supplemental Data 5**. The heatmap shows relative proportion of FPKM values (%) based on sulfur and nitrogen genes linked to the top hit taxonomical affiliation in the UniProtKB/Swiss-Prot database. Family level of is shown for most taxa, while some gene-taxa affiliations could only be linked to class or unclassified bacteria (shown in turquoise text). A separate Excel file has been uploaded.

**Supplemental Table 1.** The first table shows sequence data from the 16S rRNA gene dataset of the amount of raw read pairs, reads after merging, quality trimming, and the amount of clustered OTUs. The second table shows the amount of raw read pairs from the RNA dataset, contigs after assembly, and amount of annotated and unclassified transcripts.

**Supplemental Table 2.** Chemistry data from the water phase and in the top 1 cm sediment slices from the field and incubation experiment (SD = 1). Replicates are denoted as *n* in the table. All sediment chemistry measurements were conducted on 0.7 µm filtered pore-water except oxygen depth (mm distance in the sediment surface) and percentage of organic matter (dried sediment). Oxygen values are from Broman et al (2017).

**Reference**

Broman E, Varvara S, Dopson M, Hylander S (2017). Diatoms dominate the eukaryotic metatranscriptome during spring in coastal ‘dead zone’ sediments. *Proc R Soc Lond B Biol Sci***:** In press.

**Supplemental Table 3**. Alpha diversity (Shannon H) index values for the water and sediment samples. OTU counts were sub sampled to the lowest sample size and bootstrapped 100 times before the index was calculated, SD = 1.

**Supplemental Table 4**. Pearson correlations of the top 100 abundant OTUs (viable cells) in the water phase and oxygen concentrations from both treatments (oxic and anoxic) of all time points (field and experiment; *n* = 12). Only significant correlations are shown. P values < 0.01 are denoted as ** while correlations with a P < 0.05 are denoted *. Positive significant correlations are shown with the red gradient while negative significant correlations have a blue gradient.

**Supplemental Table 5**. Pearson correlations of chemistry and OTU data of viable cells from both treatments (oxic and anoxic) at all time points. The dataset has been divided into water (*n* = 14) and sediment (*n* = 16) and grouped by phylum, class, and genus. The water data had a total of 38 phyla, 66 classes, and 209 genera; while the sediment data had a total of 37 phyla, 62 classes, and 188 genera. From this data only statistically significant changes are shown. Values with a p < 0.01 are denoted by a solid purple border while correlations with a p < 0.05 are denoted by a dashed light purple border. Positive significant correlations are denoted by a black background and white text while negative significant correlations are denoted by a light gray background.

**
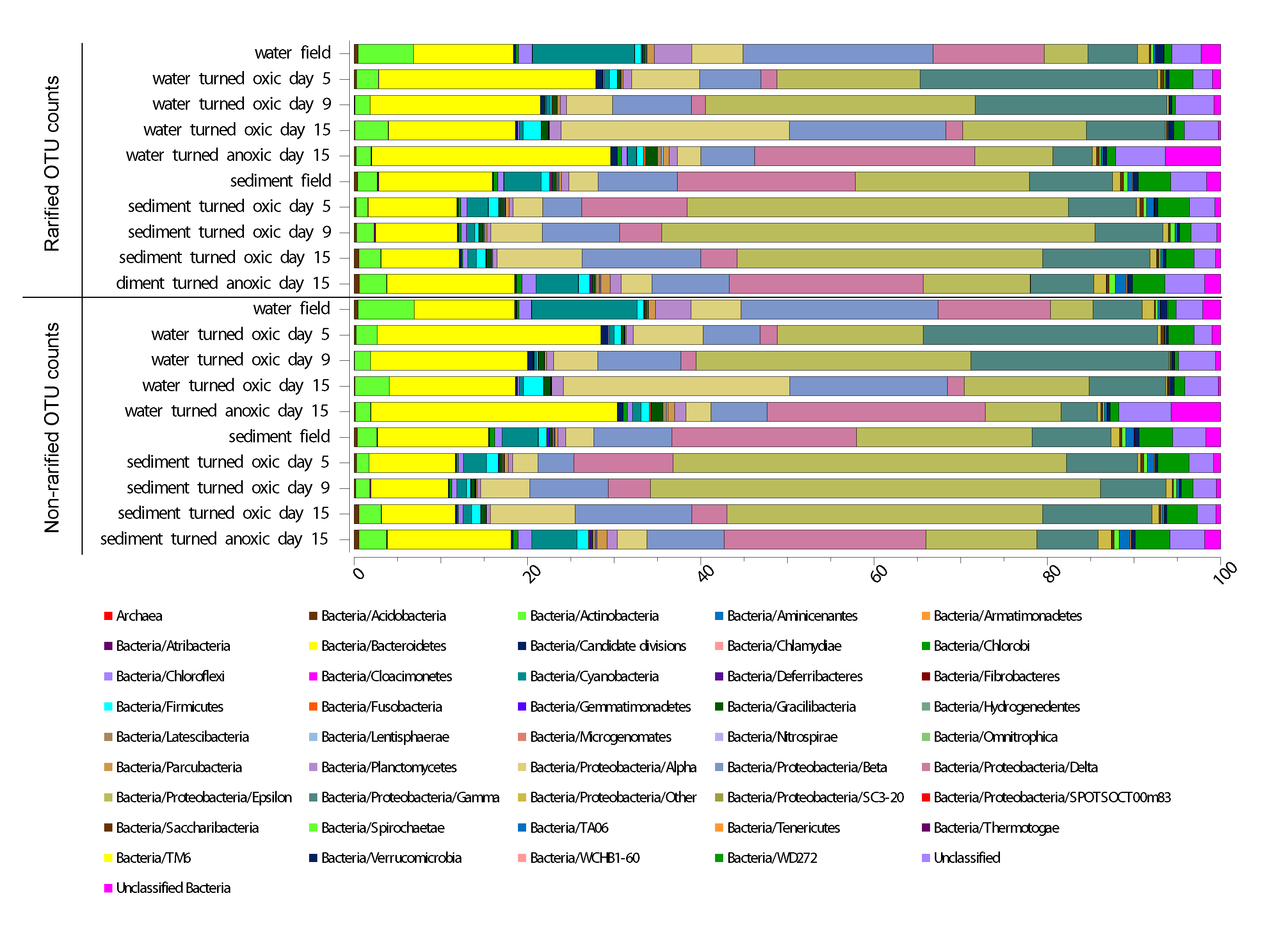
**

**Supplemental Figure 1.** Stacked bar graphs of partial 16S rRNA gene sequences from the water phase and the sediment surface (top 1 cm) collected in the field and throughout the incubation experiment. Averages of relative abundances of rarefied OTU counts are shown for the viable cells (PMA-treated) and relative abundance of non-rarefied OTU counts. OTU counts ranged from 1776 to 49665 and the dataset was rarefied to the lowest sample size. Proteobacteria have been divided into classes.


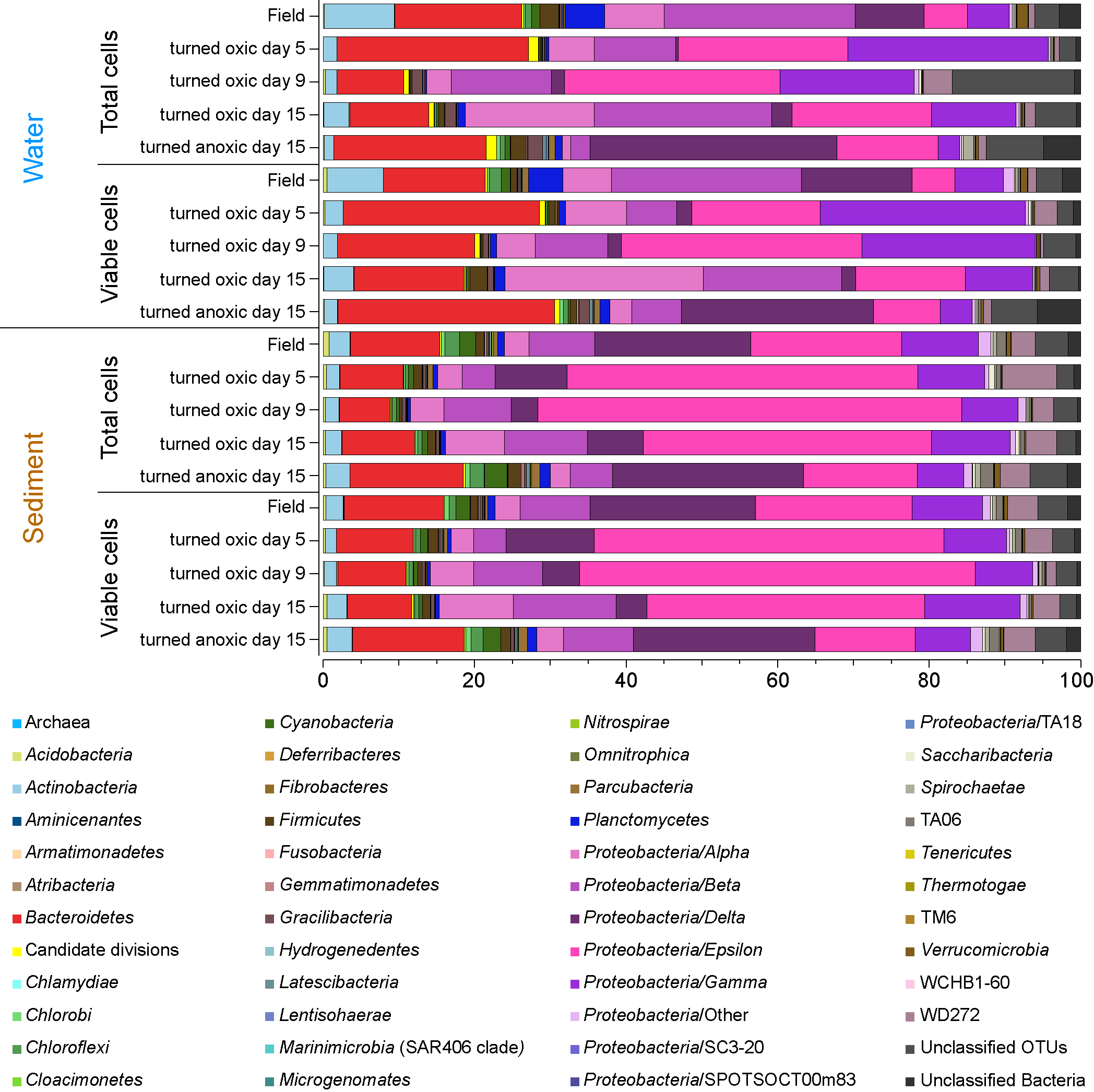


**Supplemental Figure 2.** Stacked bar graphs of partial 16S rRNA gene sequences from the water phase and the sediment surface (top 1 cm) collected in the field and throughout the incubation experiment. Averages of relative abundances are shown. Both total and viable cells (PMA-treated) are shown. Proteobacteria have been divided into classes.


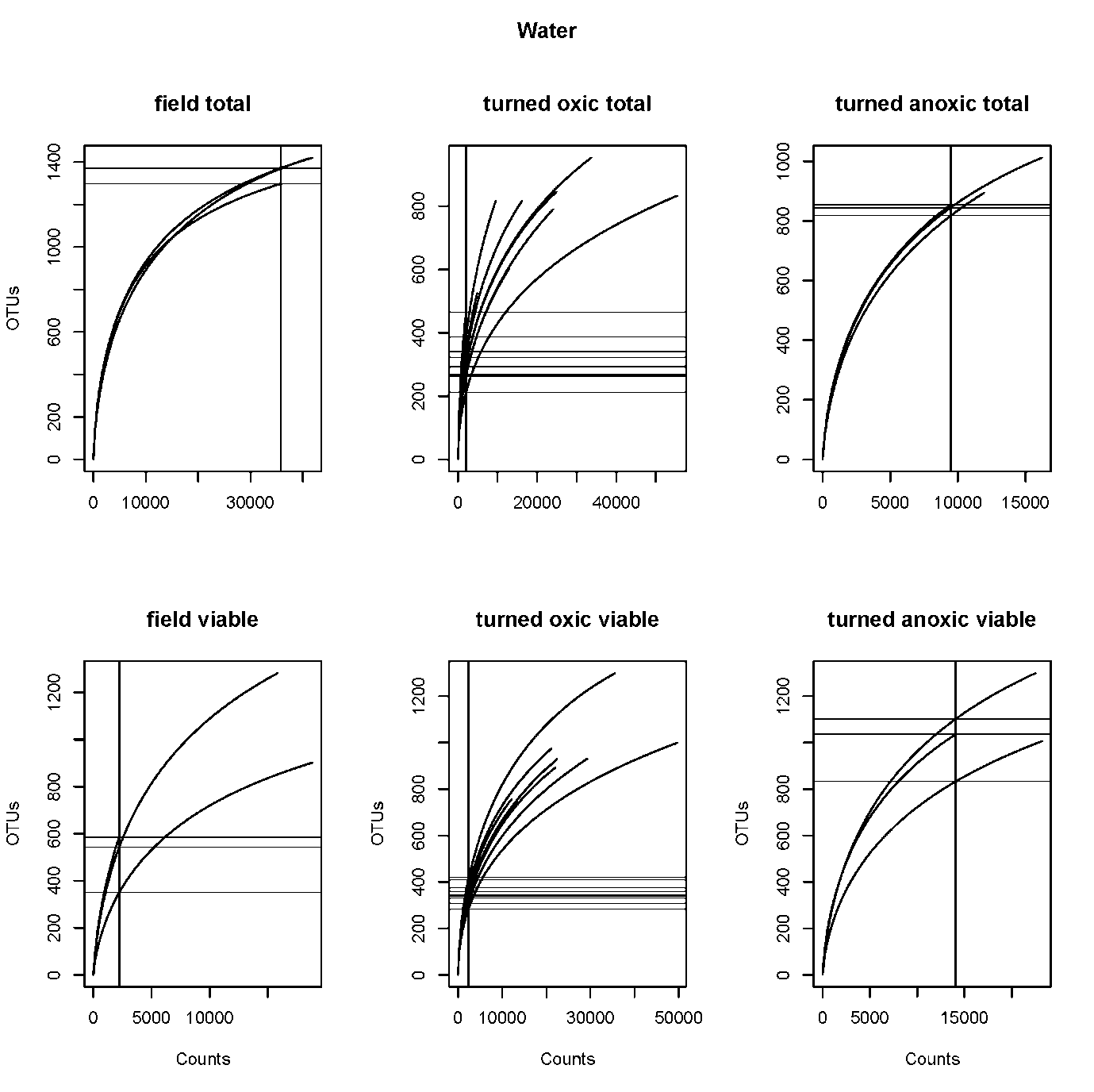


**
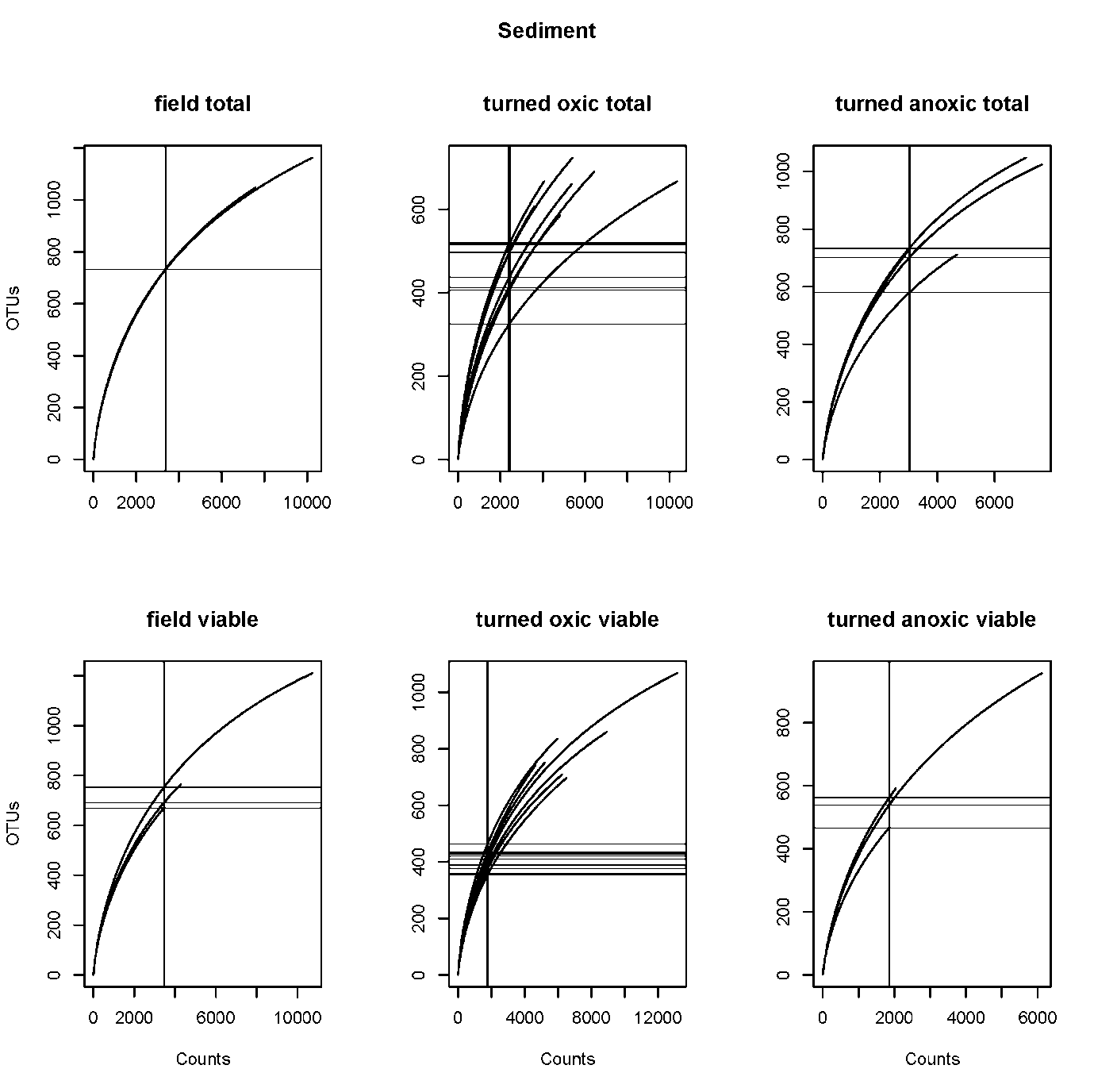
**

**Supplemental Figure 3.** Water and sediment rarefaction curves of OTUs and read counts from the final quality trimmed reads used to cluster OTUs. The lowest sample sizes were calculated and are indicated by the horizontal lines in the plots (subsampled species richness). Vertical lines indicate were the samples cross the horizontal line.

**
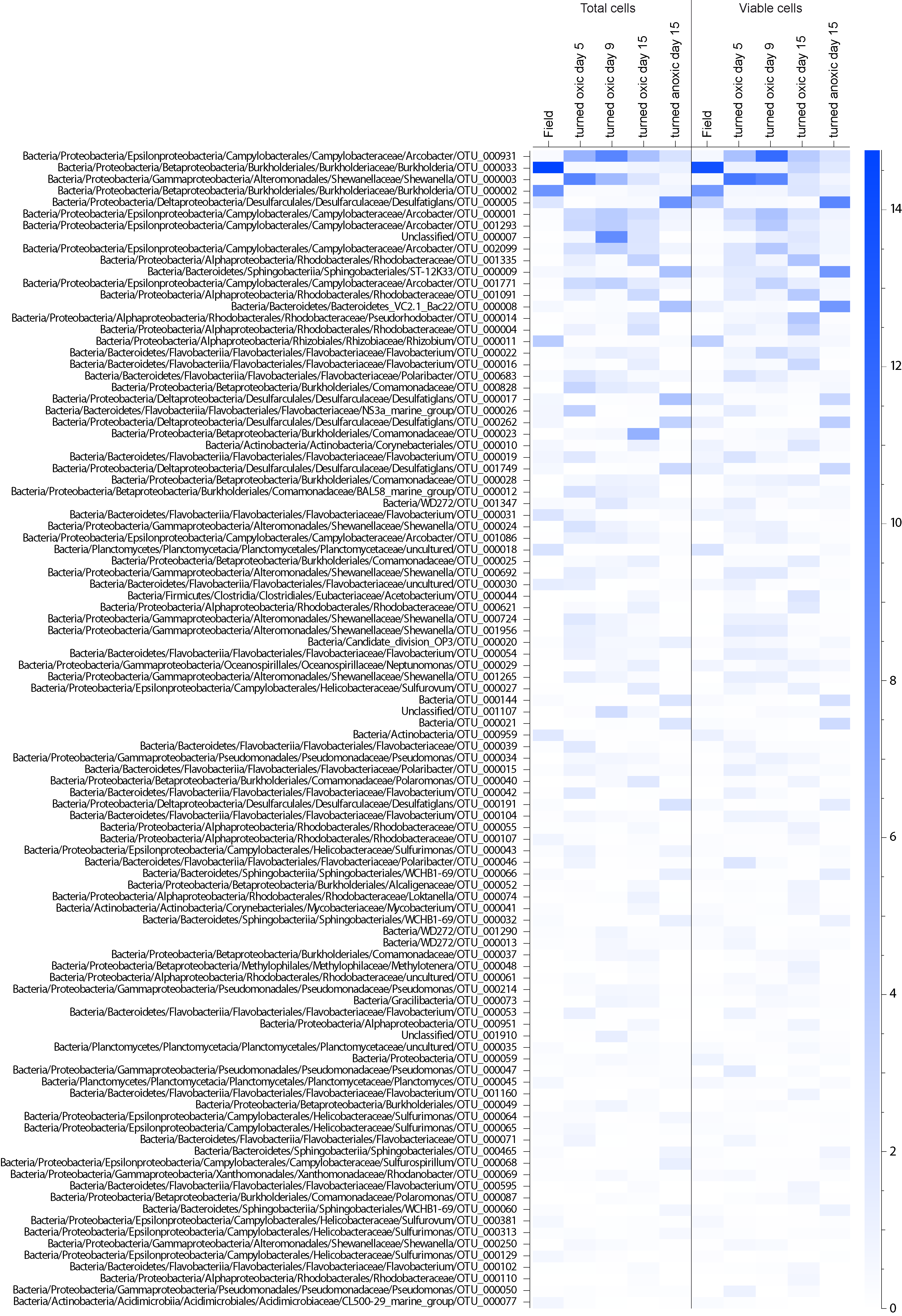
Supplemental Figure 4**. Top 100 most relatively abundant total and viable OTUs in the water phase (blue heatmap). Samples consist of biological triplicates except ‘turned oxic’ day 9 (*n* = 2) and ‘turned oxic’ day 15 (*n* = 4).

**
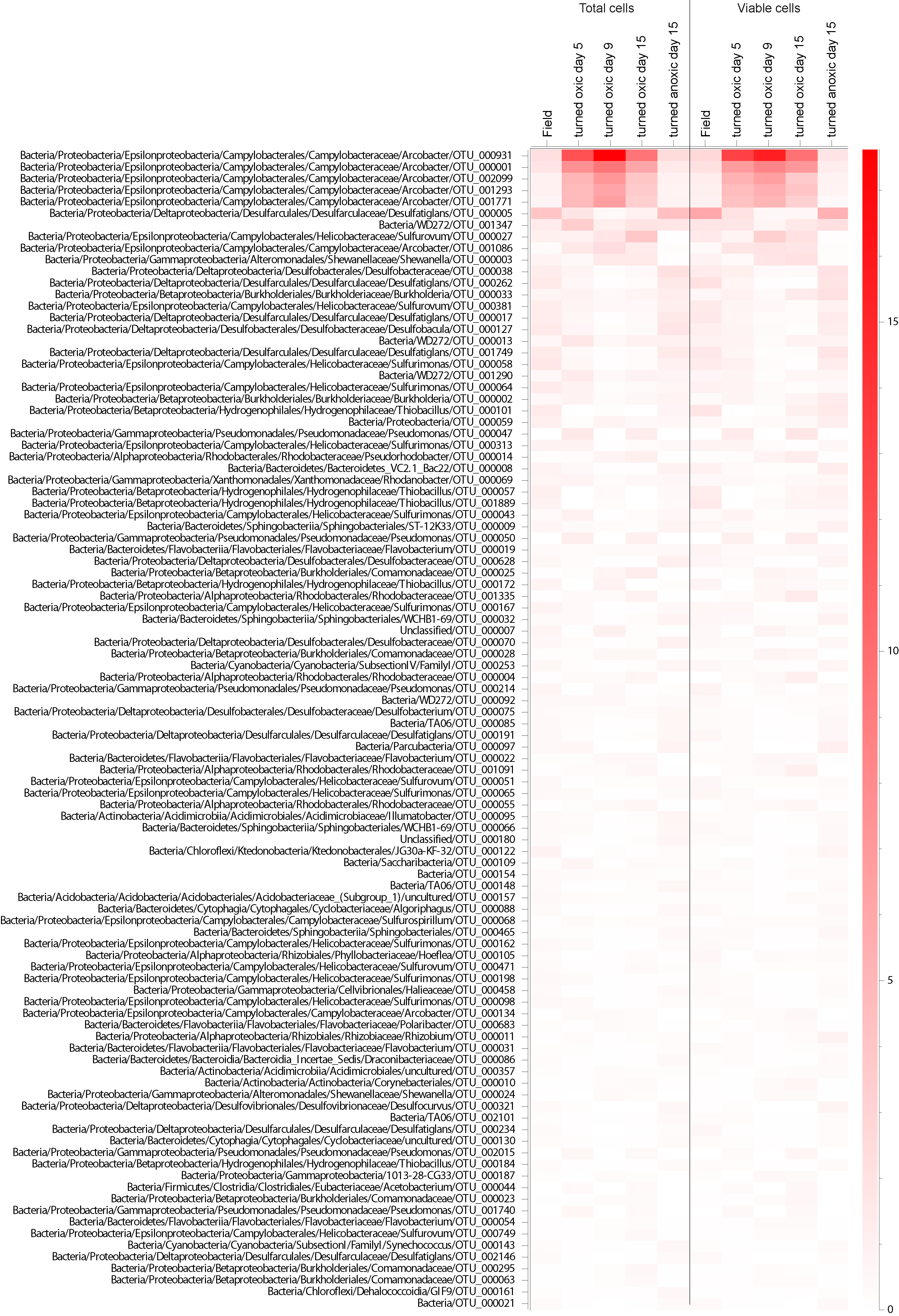
**

**Supplemental Figure 5**. Top 100 most relative abundant total and viable OTUs in the top 1 cm sediment surface (red heatmap). Samples consist of biological triplicates except ‘turned oxic’ day 15 (*n* = 4).

**Supplemental Figure 6**. Correlation network of statistically significant sediment *Arcobacter* OTUs interactions with other OTUs. The dataset was delimited to correlation coefficients: r < -0.7 or > 0.7, and a statistical significance of p < 0.01. Node names are denoted by the deepest taxonomic annotation followed by a specific OTU number. Line colors represent the range of the correlation coefficient, with red: r < -0.7, and light green r > 0.7. A separate .pdf file has been uploaded.
